# An orthotropic electro-viscoelastic model for the heart with stress-assisted diffusion

**DOI:** 10.1007/s10237-019-01237-y

**Published:** 2019-10-19

**Authors:** Adrienne Propp, Alessio Gizzi, Francesc Levrero-Florencio, Ricardo Ruiz-Baier

**Affiliations:** 1 Mathematical Institute, University of Oxford, A. Wiles Building, Woodstock Road, Oxford, OX2 6GG United Kingdom; 2 Nonlinear Physics and Mathematical Modeling Laboratory, Department of Engineering, University Campus Bio-Medico, Rome, Italy; 3 Department of Computer Science, University of Oxford, 15 Parks Road, Oxford, OX1 3QD United Kingdom; 4 Laboratory of Mathematical Modelling, Institute of Personalised Medicine, Sechenov University, Moscow, Russian Federation

**Keywords:** Orthotropic nonlinear elasticity, Mixed-primal finite element method, Kirchhoff stress formulation, Stress-assisted diffusion, Viscoelastic response, Cardiac electromechanics, 65M60, 92C10, 74S05, 74F99, 74D10

## Abstract

We propose and analyse the properties of a new class of models for the electromechanics of cardiac tissue. The set of governing equations consists of nonlinear elasticity using a viscoelastic and orthotropic exponential constitutive law, for both active stress and active strain formulations of active mechanics, coupled with a four-variable phenomenological model for human cardiac cell electrophysiology, which produces an accurate description of the action potential. The conductivities in the model of electric propagation are modified according to stress, inducing an additional degree of nonlinearity and anisotropy in the coupling mechanisms, and the activation model assumes a simplified stretch–calcium interaction generating active tension or active strain. The influence of the new terms in the electromechanical model is evaluated through a sensitivity analysis, and we provide numerical validation through a set of computational tests using a novel mixed-primal finite element scheme.

## Introduction

In order to effectively combat cardiovascular disease, we need a robust scientific understanding of the mechanisms of the heart and the nature of such health conditions. Recent progress in the field is encouraging; the concept of patient-specific treatment is no longer a distant dream, but a conceivable reality and a topic of ongoing research. However, a major difficulty is our incomplete knowledge about the relationship between processes at the cellular and sub-cellular level, and the performance of the organ as a whole (Augustin et al. 2016). Indeed, a great deal of treatment is still based on trial-and-error experimentation rather than a more fundamental scientific understanding of the changes responsible for the onset and progression of disease (Göktepe et al. 2013). Several treatments, such as resynchronisation therapy and anti-arrhythmic medications, for example, are known to be ineffective or even exacerbate pathological conditions in some patients, for reasons that are not yet well understood (Jaffe and Morin 2014). One potential obstacle to deep understanding of cardiac function is the difficulty of acquiring sufficiently detailed data. Until recently, there were no experimental techniques capable of recording 3D cardiac activity with high enough spatiotemporal resolution to provide the required level of information. However, relatively recent studies (see, for example, Christoph et al. 2018) have used optical mapping to assess electromechanical waves with acceptable physiological accuracy.

Computational models have thus been critical in allowing for extensive study of the heart even without sufficient data. The development of complex multiscale and multiphysics models, accompanied by advances in simulation and imaging techniques, has enabled researchers to investigate the many different aspects of cardiac function and disease. The hope is that the knowledge gained from these models can contribute to new and improved treatment methods. Even though the problem of cardiac electromechanics has been the focus of a large number of modelling and computational studies (see, for instance, Augustin et al. 2016; Cherubini et al. 2012; Franzone et al. 2016; Costabal et al. 2017; Gizzi et al. 2015; Göktepe et al. 2013; Nobile et al. 2012; Quarteroni et al. 2017; Sundnes et al. 2014 and the references therein), there still remain many challenges in the development of more accurate and detailed models and the accompanying methods.

In such a context, the large majority of the proposed approaches rely on continuum formulations of the complex microstructural interactions occurring among the heart tissue components, e.g. cardiomyocytes, involving different scales (Quarteroni et al. 2017). The study of single cell and cell–cell (Lenarda et al. 2018) chemomechanical and electromechanical interactions has attempted to unveil some of the underlying complex features of the cardiac function, and different multifield nonlinear models have been gradually generalising classical approaches such as the monodomain equations and Fick’s law of diffusion. In particular, fractional diffusion (Cusimano et al. 2018), nonlinear diffusion (Hurtado et al. 2016), and stress-assisted diffusion formulations (Cherubini et al. 2017) were recently proposed to reproduce porous multiscale excitation phenomena within the framework of homogenised models for cardiac tissue. These studies, in fact, paved the route towards new challenging theoretical and computational problems aiming at a reliable in silico prediction of heart rate variability, cardiac repolarisation, and inducibility of life-threatening arrhythmias (Phadumdeo and Weinberg 2018). At the same time, macroscale incompressibility, orthotropic, and hysteretic mechanical features have been shown to fully characterise the human cardiac tissue under multiaxial loading tests (see, for example, Gültekin et al. 2016 and references therein). Viscosity properties, in particular, have been incorporated as one spring element coupled with Maxwell elements in parallel endowing the model with hysteretic characteristics describing the viscous response due to matrix, fibre, sheet, and fibre–sheet couplings through four dedicated dashpots (Gültekin et al. 2016). Also in this case, a porous medium motivation has been advanced in Yao et al. (2012), including the extracellular fluid filtrating through the elastic body, contributed by the active contractile behaviour of the muscle. However, complete agreement concerning the specific multiscale features involved in energy dissipation for the cardiac tissue, and soft biological tissues in general, is still lacking. The stress evolution equations for time-dependent viscous behaviour are based on finite-strain viscoelasticity (Holzapfel and Gasser 2001), motivated by a rheological analogue from Simo 1987, and endowed with equilibrium and non-equilibrium contributions (Lubliner 1985) in which the usual assumption of volume-preserving deformations during time-dependent responses is made.

A distinguishing feature of our approach is the introduction of the mechanoelectrical feedback (MEF) in the electric conductivities, through a direct dependence on the Kirchhoff stress. This framework, known as stress-assisted diffusion (SAD), is widely employed in the modelling of gels and polymers (Klepach and Zohdi 2014), but has only recently been adapted for active biological media undergoing reaction–diffusion excitation (Cherubini et al. 2017), and more tailored for cardiac models in Loppini et al. (2018). While these contributions consider hyperelastic formulations coupled with multiphysics activation mechanisms, we also consider here the viscoelastic effects typical of soft microstructured fibre-reinforced biological tissues, and using realistic ventricular geometries. Fully mixed methods for the hyperelasticity of the cardiac tissue (that is, formulations involving stresses or strains in addition to simply displacement or displacement–pressure) are not yet widely employed. They have been introduced in Ruiz-Baier (2015) and used more recently in Cherubini et al. (2017), Garcia-Blanco et al. (2019), and Ruiz-Baier et al. (2019). In the present case, our model and our numerical method include a three-field elasticity formulation (variationally based on a modification of the Hu–Washizu principle (Lamichhane et al. 2006)) that states the governing equations in terms of stress–displacement–pressure, motivated by the desire to avoid volumetric locking and to solve directly for additional variables of interest. In particular, we solve for the Kirchhoff stress, which we use explicitly in our incorporation of SAD. This formulation includes a pressure stabilisation term needed, in the lowest-order case, for triangular or for tetrahedral meshes. It constitutes a generalisation of the three-field formulation for nearly incompressible hyperelasticity, designed in Chavan et al. (2007) using quadrilateral meshes. Another difference in the present contribution is that we employ a more accurate cellular model, tailored for recovering human action potential dynamics, restitution features under constant pacing as well as sustained fibrillation behaviours and spiral waves breakup (Bueno-Orovio et al. 2008). While the active strain approach is adopted in many instances in the literature and is often favoured due to the practicality of measuring strains directly using imaging techniques (Rossi et al. 2014), the active stress approach is somewhat simpler and more naturally incorporated in already existing models for passive deformation (Giantesio et al. 2019). In this work, we will adopt both formulations, although we find that the active strain formulation better reproduces physiologically accurate deformation regimes in ventricular geometries. To the best of our knowledge, no previous attempts have been made incorporating both active stress and active strain within a generalised stress-assisted reaction–diffusion formalism and embedding orthotropy, incompressibility and viscoelasticity for human cardiac ventricular domains.

This paper has been structured in the following manner. Section 2 lays out the elements of the mathematical model that describes the electro-viscoelastic function of the heart, including the active contraction of the cardiac muscle and the representation of the mechanoelectric feedback using stress-assisted conductivity, as well as a contribution from geometric nonlinearities (or geometrical feedback). The passive hyperelastic response of the tissue is described by an orthotropic exponential model, whereas the ionic activity which causes active contraction is incorporated through orthotropic active stress. Active strain will also be addressed. The specific structure of the governing equations (written in terms of stress, displacements, electric potential, activation generation, and ionic variables) suggests to cast the problem in a mixed-primal form, and to use a mixed-primal finite element method for its numerical approximation. This is precisely the method that we outline in Sect. 3, which also includes a description of the consistent linearisation and implementation details. Our computational results in 2D and 3D, along with numerical validation and pertinent discussions on the modelling considerations, are then presented in Sect. 4. We close with a summary and some remarks on model limitations and ongoing extensions, collected in Sect. 5.

## Mathematical model

### Finite-strain cardiac mechanics

Let $\varOmega \subset \mathbb {R}^d$, $d\in \{2,3\}$, denote a deformable body with a piecewise smooth boundary $\partial \varOmega$, considered in its reference configuration, and let $\varvec{n}$ denote the outward unit normal vector on $\partial \varOmega$. The kinematical description of finite deformations regarded on a time interval $t\in (0,t_{\text {final}}]$ is made precise as follows. A material point in $\varOmega$ is denoted by $\varvec{x}$, whereas $\varvec{u}(t):\varOmega \rightarrow \mathbb {R}^d$ will denote the displacement field defining its new position in the deformed configuration. The tensor $\mathbf {F}:=\mathbf {I}+ \nabla \varvec{u}$ is the gradient (applied with respect to the fixed material coordinates) of the deformation map; its determinant, denoted by $J=\det \mathbf {F}$, measures the solid volume change during the deformation; and $\mathbf {C}=\mathbf {F}^\mathtt{t}\mathbf {F}$ and $\mathbf {B}=\mathbf {F}\mathbf {F}^\mathtt{t}$ are, respectively, the right and left Cauchy–Green deformation tensors on which all strain measures will be based (here the superscript $()^\mathtt{t}$ denotes the transpose operator). The first isotropic invariant ruling deviatoric effects is the scalar $I_{1}(\mathbf {C})=\mathrm{tr} \, \mathbf {C}$, and for generic unitary vectors ${\varvec{f}_0}, {\varvec{s}_0}$, the scalars $I_{4,f}(\mathbf {C})={\varvec{f}_0}\cdot (\mathbf {C}{\varvec{f}_0})$, $I_{4,s}(\mathbf {C})={\varvec{s}_0}\cdot (\mathbf {C}{\varvec{s}_0})$, $I_{8,fs} (\mathbf {C})={\varvec{f}_0}\cdot (\mathbf {C}{\varvec{s}_0})$ are pseudo-invariants of $\mathbf {C}$ measuring direction-specific stretch (Ciarlet 1988).

The triplet $({\varvec{f}_0}(\varvec{x}),{\varvec{s}_0}(\varvec{x}),{\varvec{n}_0}(\varvec{x}))$ represents an orthogonal coordinate system pointing in the local direction of the muscular cardiac fibres, transversal sheetlet compound, and normal cross-fibre direction ${\varvec{n}_0}(\varvec{x}) = {\varvec{f}_0}(\varvec{x})\times {\varvec{s}_0}(\varvec{x})$. Note that the system is restricted to $({\varvec{f}_0}(\varvec{x}),{\varvec{s}_0}(\varvec{x}))$ in the two-dimensional case, and that these directions are defined in the reference configuration. Constitutive relations characterising the material properties and underlying microstructure of the myocardial tissue will follow the orthotropic model proposed in Holzapfel and Ogden (2009), whose strain energy density (relating the amount of energy stored within the material in response [joule/volume] to strain, and which assumes an additive decomposition into isotropic, volumetric, and anisotropic contributions) and the first Piola–Kirchhoff stress tensor (associated with a passive, elastic deformation) read, respectively

2.1 $$\begin{aligned} \varPsi _{\mathrm {pas}}(\mathbf {F})&= \frac{a}{2b}e^{b(I_{1}-d)} + \sum _{i\in \{ f,s\} } \dfrac{a_{i}}{2b_{i}}\bigl [e^{b_{i}(I_{4,i}-1)_+^{2}}-1\bigr ] \\&\qquad +\dfrac{a_{fs}}{2b_{fs}}\bigl [e^{b_{fs} (I_{8,fs})^2 } -1\bigr ], \\ \mathbf {P}_{\mathrm {pas}}&=\frac{\partial \varPsi _{\mathrm {pas}}}{\partial \mathbf {F}}-pJ\mathbf {F}^{-\mathtt{t}}, \end{aligned}$$

where *a*, *b* are material constants associated with the isotropic matrix response, $a_f$ and $b_f$ rule the directional behaviour of the material along myocardial fibres, $a_s$ and $b_s$ account for the cross-contribution of the fibre–sheet directions, and $a_{fs},b_{fs}$ encapsulate the shear effects in the fibre–sheet plane. Moreover, the field *p* denotes the solid hydrostatic pressure, and we use the notation $(u)_+:= u$ if $u>0$ or zero otherwise, for a generic real-valued function *u*. This modelling choice is appropriate given that fibres have a quite different behaviour under compression or tension regimes. In addition, taking the positive part of the exponents in the anisotropic energy results in excluding anisotropic energetic contributions for compressed fibre configurations, which in the case of passive fibres should have an effect only during extension (Pezzuto et al. 2014). We remark here that the particular mechanisms of soft tissue anisotropic mechanical behaviour are still under investigation (Humphrey et al. 2014), and the chosen formulation may not be the most general one. Moreover, full incompressibility of the tissue will be enforced in the present framework, and this has some advantages associated with the mathematical and numerical structure of the system. Although biological tissues possess a complex porous structure, compression features are still being systematically investigated ex vivo, and a more comprehensive answer on the subject is still needed (McEvoy et al. 2018).

### Active stress and active strain

In physiological scenarios, the mechanical deformation is also actively influenced by microscopic tension generation.

*Active stress model.* A simple description is given in terms of active stresses (see, for instance, Sundnes et al. 2014): we assume that the first Piola-Kirchhoff stress tensor decomposes as

2.2 $$\begin{aligned} \mathbf {P}= \mathbf {P}_{\mathrm {pas}} + \mathbf {P}_{\mathrm {act}}, \end{aligned}$$

where the active stress component acts differently on each local direction with an intensity depending on the scalar field of active tension $T_a$, that synthesises (in an homogenised sense) the biochemical state of myocytes (and whose dynamic behaviour will be specified later on). Then,

2.3 $$\begin{aligned} \mathbf {P}_{\text {act}}&= J\varvec{\sigma }_{\text {act}} \mathbf {F}^{-\mathtt{t}}, \quad \text {with} \\ \varvec{\sigma }_{\text {act}}&= \frac{T_a}{J\lambda _f} \mathbf {F}{\varvec{f}_0}\otimes \mathbf {F}{\varvec{f}_0}+ \frac{\kappa _{sn} T_a}{J\lambda _s\lambda _n} \mathrm {sym}(\mathbf {F}{\varvec{s}_0}\otimes \mathbf {F}{\varvec{n}_0}) \\&\quad +\frac{\kappa _{nn}T_a}{J\lambda _n} \mathbf {F}{\varvec{n}_0}\otimes \mathbf {F}{\varvec{n}_0}, \end{aligned}$$

where $\kappa _{sn},\kappa _{nn}$ are positive constants representing the variation of activation on each specific direction, as proposed in Dorri et al. (2006), and $\lambda _f=\sqrt{I_{4,f}},\lambda _s=\sqrt{I_{4,s}},\lambda _n=\sqrt{{\varvec{n}_0}\cdot (\mathbf {C}{\varvec{n}_0})}$ are the fibre, sheetlet, and cross-fibre stretches. Setting appropriate models for $\varvec{\sigma }_{\text {act}}$ is not a trivial task since the active contribution to the force should account for the geometric properties of deformation, and these undergo substantial changes during contraction in the finite-strain regime (Pezzuto et al. 2014). Details of other anisotropic activation forms can be found, for instance, in Rossi et al. (2014) for active strain and in (Usyk et al. 2000, Appendix B) for active stress descriptions, but they are basically responsible for additional deformation effects such as wall thickening, radial constriction and torsion, as well as longitudinal shortening. Note that the active Cauchy stress does not include a contribution on the diagonal entry associated with the local sheetlet direction ${\varvec{s}_0}$ since a stress component on this direction would counteract wall thickening mechanisms (Dorri et al. 2006). Moreover, the intensity of the active tension effect on the cross-fibre direction ${\varvec{n}_0}$ is assumed to be substantially smaller than that appearing on the off-diagonal component $\mathrm {sym}(\mathbf {F}{\varvec{s}_0}\otimes \mathbf {F}{\varvec{n}_0})$; see Table 2. Also note that some references do not include a normalisation with local stretches in each term of $\varvec{\sigma }_{\text {act}}$. Finally, these constitutive laws are usually not derived from a thermodynamic potential.

*Active strain model.* Next we recall the active strain model for ventricular electromechanics (see, for example, Cherubini et al. 2008). The contraction of the tissue results from activation mechanisms governed by internal variables and incorporated into the finite elasticity context using a multiplicative decomposition of the deformation gradient into a passive (purely elastic) and an active part, $\mathbf {F}=\mathbf {F}_{E}\mathbf {F}_{A}$, with

2.4 $$\begin{aligned} \mathbf {F}_{A}&=\mathbf {I}+\gamma _f{\varvec{f}_0}(\varvec{x})\otimes {\varvec{f}_0}(\varvec{x}) \\&\qquad +\gamma _s{\varvec{s}_0}(\varvec{x}) \otimes {\varvec{s}_0}(\varvec{x})+\gamma _n{\varvec{n}_0}(\varvec{x})\otimes {\varvec{n}_0}(\varvec{x}). \end{aligned}$$

The coefficients $\gamma _i$, with $i=f,s,n$, are smooth scalar functions encoding the macroscopic stretch in specific directions, whose precise definition will be postponed to (2.15). The inelastic contribution to the deformation modifies the length and potentially also the shape of the cardiac fibres, and then, compatibility of the motion is restored through an elastic deformation accommodating the active strain distortion. A physiological motivation for the active strain approach is related to the shortening of sarcomeres as a response to the sliding filaments of the actin–myosin molecular motor: such shortening is encapsulated in $\mathbf {F}_{A}$, which determines a new (and fictitious, or virtual) intermediate configuration that is regarded as a reference for the elastic deformation (Pezzuto et al. 2014). Therefore, the strain energy function and the first Piola-Kirchhoff stress tensor (after applying the active strain decomposition) are functions of $\mathbf {F}_{E}$ only, and read, respectively

2.5 $$\begin{aligned} {\widehat{\varPsi }}(\mathbf {F}_E)&= \frac{a}{2b}e^{b(I^E_{1}-d)} + \sum _{i\in \{ f,s\} } \dfrac{a_{i}}{2b_{i}}\bigl [e^{b_{i}(I^E_{4,i}-1)_+^{2}}-1\bigr ] \\&\quad +\dfrac{a_{fs}}{2b_{fs}}\bigl [e^{b_{fs}(I_{8,fs}^E)^{2}}-1\bigr ] , \\ \mathbf {P}&=\frac{\partial {\widehat{\varPsi }}}{\partial \mathbf {F}}-pJ\mathbf {F}^{-\mathtt{t}}. \end{aligned}$$

As in the description of (2.1) above, we again note that one switches off the anisotropic contributions under compression. An additional advantage is that the associated terms in the strain energy function (in both the pure passive and active strain formulations) can be shown to be strongly elliptic (Pezzuto et al. 2014) (these will be the terms appearing on the second diagonal block of the weak formulation from Sect. 3, the block corresponding to displacements); however, the overall problem will remain of a saddle-point structure. The modified elastic invariants $I_i^E$ are functions of the coefficients $\gamma _i$, as well as of the invariant and pseudo-invariants in the following manner (Rossi et al. 2012)

$$\begin{aligned} I_{1}^{E}&= I_1 - \gamma _{f}\frac{\gamma _{f}+2}{(1+\gamma _{f})^2} I_{4,f} - \gamma _{s}\frac{\gamma _{s}+2}{(1+\gamma _{s})^2} I_{4,s} \\ {}&- \gamma _{n}\frac{\gamma _{n}+2}{(1+\gamma _{n})^2} I_{4,n} \,, \\ I_{4,f}^E&= \left( 1+\gamma _{f}\right) ^{-2} I_{4,f} ,\qquad I_{4,s}^E = \left( 1+\gamma _{f}\right) I_{4,s},\\ I_{8,fs}^E&= \left( 1+\gamma _{f}\right) ^{-1/2} I_{8,fs}\,. \end{aligned}$$

Such dependencies are a consequence of assuming isochoric active deformations (Pezzuto et al. 2014), i.e. $\det \mathbf {F}_{A}=1$, justified by the fact that the volume of the cardiomyocytes does not vary substantially during contraction. Besides, following Rossi et al. 2012, previous expressions are obtained by assuming $\gamma _s=\gamma _n$ and making use of the fact that $I_1=I_{4,f}+I_{4,s}+I_{4,n}$, as well as that $\mathbf {F}_{E}=\mathbf {F}\mathbf {F}_{A}^{-1}$, with

$$\begin{aligned} \det \mathbf {F}_{A}&= (1+\gamma _f)(1+\gamma _s)(1+\gamma _n) \,, \\ \mathbf {F}_{A}^{-1}&= \mathbf {I}- \dfrac{\gamma _f}{1+\gamma _f} {\varvec{f}_0}\otimes {\varvec{f}_0}\\ {}&- \dfrac{\gamma _s}{1+\gamma _s} {\varvec{s}_0}\otimes {\varvec{s}_0}- \dfrac{\gamma _n}{1+\gamma _n} {\varvec{n}_0}\otimes {\varvec{n}_0}\,. \end{aligned}$$

Accordingly, the active strain, and consequently the force associated with the active part of the total stress, will receive contributions acting on the three main directions. The calcium-based activation signal travels up to four times faster along the fibre axis than in the sheet and normal directions, and this fact further motivates the use of orthotropic active strain (Rossi et al. 2014).

### Viscoelasticity and equations of motion

Extension and shear tests demonstrate the importance of incorporating viscoelastic effects in models for cardiac passive mechanics (Gültekin et al. 2016). In the heart, the extracellular fluid filtrating through the elastic solid is one of the main generators of the viscoelastic effects of the tissue (Yao et al. 2012). Viscous effects are also tied to cross-bridge processes identified in Ca$^{2+}$ activated fibres (Maughan et al. 1998) and have a well-established literature as well as a consistent methodology for their implementation (the stress update algorithm that uses a convolution integral representation) developed for general soft tissues (Holzapfel and Gasser 2001). From the viewpoint of kinematics, it suffices to relate stress to strain rates. Decomposition of the spatial velocity gradient $\varvec{w}= \dot{\varvec{u}}$ into the rate of deformation and spin tensors yields the relation

$$\begin{aligned} \dot{\mathbf {B}} = \nabla \varvec{w}\mathbf {B}+ \mathbf {B}(\nabla \varvec{w})^\mathtt{t}, \end{aligned}$$

and a simplified rheological Kelvin–Voigt model for the viscous component of the Cauchy stress can be defined as follows (see, for example, Karlsen 2017):

2.6 $$\begin{aligned} \varvec{\sigma }_{\text {visc}} = \delta e^{\beta \dot{I_1}} \dot{\mathbf {B}}, \end{aligned}$$

which depends on the history of the isotropic contribution to the Cauchy stress. Here, $\delta ,\beta >0$ are model parameters. In this way, after a pull-back operation, we see that

2.7 $$\begin{aligned} \mathbf {P}_{\text {tot}} = \mathbf {P}+ J\varvec{\sigma }_{\text {visc}} \mathbf {F}^{-\mathtt t}, \end{aligned}$$

is the total first Piola-Kirchhoff stress tensor that includes $\mathbf {P}$ defined from either (2.1)–(2.2) or (2.5), and the viscoelastic contributions.

More advanced rheologies can be easily incorporated in the context of active stress formulations as done in, e.g. (Katsnelson et al. 2004), as the generalised Hill–Maxwell model recently proposed in Cansiz et al. 2017, as in the perturbed equations of harmonic wave motion using springpot-based models with fractional order derivatives from Capilnasiu et al. 2019, or as in the thermodynamical electro-viscoelastic models that use statistical fibre distributions (Pandolfi et al. 2017; Gizzi et al. 2018). We will, however, confine the presentation to (2.7) without introducing stochasticity of the anisotropic components.

Irrespective of the activation formalism one adopts (active strain or active stress), the balance of linear momentum and the incompressibility constraint (allowing only isochoric motions) are written together in the following way, when posed in the inertial reference frame and under transient mechanical equilibrium,

2.8a $$\begin{aligned} \rho \partial _{tt}\varvec{u}- \varvec{\nabla }\cdot \mathbf {P}_{\text {tot}}&= \rho _0 \varvec{b}&\text {in } \varOmega \times (0,t_{\mathrm {final}}], \end{aligned}$$

2.8b $$\begin{aligned} \rho J - \rho _0&= 0&\text {in } \varOmega \times (0,t_{\mathrm {final}}], \end{aligned}$$

where $\rho _0, \rho$ are the reference and current medium density, $\varvec{b}$ is a smooth vector field of imposed body loads, $\partial _{tt}$ denotes the second time derivative, and the divergence operator in (2.8a) acts on the tensor fields row by row. The balance of angular momentum translates into the condition that the Kirchhoff stress tensor $\varvec{\varPi }= \mathbf {P}_{\text {tot}}\mathbf {F}^\mathtt{t}$ must be symmetric, which is in turn encapsulated into the momentum and constitutive relations (2.8a), (2.1), (2.5).

Defining

$$\begin{aligned} \mathcal {G}= {\left\{ \begin{array}{ll} \displaystyle \mathcal {G}(\varvec{u},T_a) &{} := \frac{\partial \varPsi }{\partial \mathbf {F}}\mathbf {F}^\mathtt{t} + J\varvec{\sigma }_{\text {visc}} + \mathbf {P}_{\text {act}}\mathbf {F}^\mathtt{t}\\ &{}\qquad \qquad \qquad \text {for active stress},\\ \displaystyle \mathcal {G}(\varvec{u},\gamma )&{} := \frac{\partial {\widehat{\varPsi }}}{\partial \mathbf {F}}\mathbf {F}^\mathtt{t} + J\varvec{\sigma }_{\text {visc}} \\ &{}\qquad \qquad \qquad \text {for active strain}, \end{array}\right. } \end{aligned}$$

as the contribution to the Kirchhoff stress that does not involve pressure, we then have

2.9 $$\begin{aligned} \varvec{\varPi }= \mathcal {G}- p J\mathbf {I}. \end{aligned}$$

Stating the balance equations in terms of Kirchhoff stress, displacements, and pressure suggests that at the level of writing finite element schemes, we will use mixed methods respecting the same structure. Additionally, setting boundary conditions for the motion of the left ventricle is not trivial, as the organ is known to slightly move and twist during the heartbeat. In our case, Eqs. (2.8a)–(2.8b)–(2.9) will be supplemented with mixed normal displacement and traction boundary conditions

2.10a $$\begin{aligned} \varvec{u}\cdot \varvec{n}&= 0&\text {on}\ \partial \varOmega _D\times (0,t_{\mathrm {final}}], \end{aligned}$$

2.10b $$\begin{aligned} \varvec{\varPi }\mathbf {F}^{-\mathtt t} \varvec{n}- p_N J \mathbf {F}^{-\mathtt t}\varvec{n}&= \varvec{0}&\text {on}\ \partial \varOmega _N\times (0,t_{\mathrm {final}}], \end{aligned}$$

**Fig. 1:**
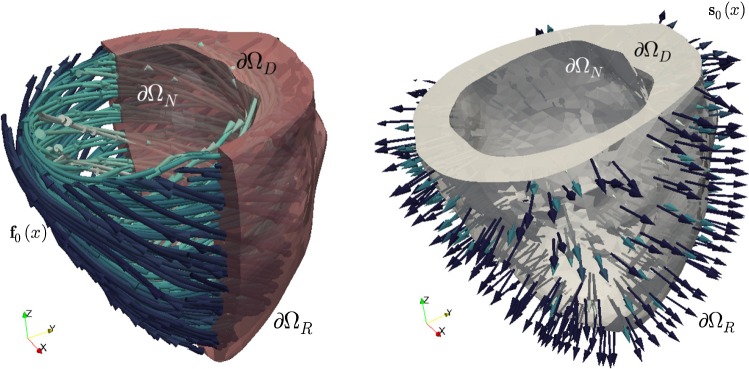
Schematic representation of a mono-ventricular domain where (2.10a) is imposed on the basal cut, (2.10b) on the endocardial surface, and (2.10c) on the epicardium. The left panel depicts the fibre field and the right panel the sheetlet directions (in this case, parallel to the normal direction of the epicardium)

2.10c $$\begin{aligned} \varvec{\varPi }\mathbf {F}^{-\mathtt t} \varvec{n}+ \eta J \mathbf {F}^{-\mathtt t} \varvec{u}&= \varvec{0}&\text {on}\ \partial \varOmega _R \times (0,t_{\mathrm {final}}], \end{aligned}$$

where $\partial \varOmega _D$, $\partial \varOmega _N$, $\partial \varOmega _R$ conform a disjoint partition of the boundary. The condition (2.10a) means that we constrain the normal motion along the normal direction with respect to the surface $\partial \varOmega _D$. The term $p_N$ in (2.10b) denotes a (possibly time dependent) prescribed boundary pressure associated with endocardial blood pressure, which is uniform over the deformed counterpart of $\partial \varOmega _N$, and it is applied in the normal direction to the epicardium in the deformed configuration. However, owing to Nanson’s formula (Ciarlet 1988), this contribution regarded on the reference configuration depends on the cofactor of the deformation gradient and therefore, the boundary condition is nonlinear in the undeformed configuration; moreover, the traction written in terms of the Kirchhoff stress tensor is $\varvec{t}= \varvec{\varPi }\mathbf {F}^{-\mathtt t}\varvec{n}$. Also note that the Robin conditions (2.10c) account for stiff springs connecting the cardiac medium with the surrounding soft tissue and organs (whose stiffness is encoded in the scalar $\eta$). More sophisticated boundary conditions that consider an interaction with the pericardium can be also imposed (Fritz et al. 2014). A sketch of a mono-ventricular domain specifying boundary surfaces and fibre directions is depicted in Fig. 1.

### Monodomain equations

In the context of electromechanical processes, the propagation of electric potential *v* is governed by the following reaction–diffusion system, known as the monodomain equations (see, for example, Franzone et al. 2014), which are cast here in the reference configuration. The current conservation is written only in terms of the transmembrane potential, and the coupling with additional ionic quantities is encoded in the vector $\vec {r}$ (here we use $\vec {\cdot }$ instead of bold to denote vector fields of dimension other than *d*)

2.11a $$\begin{aligned} \chi {\partial _t v} - \nabla \cdot \{ \mathbf {D}(v,\mathbf {F},\varvec{\varPi })\, \nabla v \}&= g(v,\vec {r}) + I_{\mathrm{ext}} \\&\quad \text {in } \varOmega \times (0,t_{\mathrm {final}}], \end{aligned}$$

2.11b $$\frac{\mathrm{d}\vec {r}}{\mathrm{d}t}= \vec {m}(v,\vec {r}) \quad \text {in } \varOmega \times (0,t_{\mathrm {final}}].$$

Here, $\chi$ is the ratio of membrane area per tissue volume, and $I_{\mathrm{ext}}$ is a spatiotemporal external stimulus applied to the medium. We will adopt the minimal model for human ventricular action potential, proposed in Bueno-Orovio et al. (2008) and fitted to capture restitution curves, conduction velocity, spiral/arrhythmic dynamics, and complex behaviour typical to nonlinear dynamical systems, used later for cardiac alternans in Gizzi et al. (2013). That model was, however, tailored originally for the case of isotropic conductivity $\mathbf {D}=D\mathbf {I}$, and so the extended fully coupled model discussed below will be able to accommodate a wider class of propagation patterns and will also constitute a generalisation over other recent models for stress-assisted diffusion (Cherubini et al. 2017; Loppini et al. 2018).

Specification of the ionic currents and gating variables can be found in Appendix 1.

Boundary and initial conditions for (2.11) correspond to

2.12a $$\begin{aligned} \mathbf {D}(v,\mathbf {F},\varvec{\varPi })\, \nabla v \cdot \varvec{n}&= 0 \qquad \text {on }\partial \varOmega \times (0,t_{\text {final}}], \end{aligned}$$

2.12b $$\begin{aligned} v(0) = 0, \quad \vec {r}&= [1,1,0] \qquad \text {in }\varOmega \times \{0\}, \end{aligned}$$

and (2.12b) can be combined with suitable initial pacing, especially needed in more complex and more physiologically accurate cell models. The minimal model, as proposed in Bueno-Orovio et al. (2008), has a heterogeneous character that we do not consider in our study. Their description contains separate parameter sets that are able to reproduce experimental results for the epicardium, mid-myocardium, and endocardium, as well as parameter sets that mimic the results of two more complicated ionic models for human ventricular cells. For simplicity (and also as a consequence of lack of personalised experimental data), we use the parameter set developed for the epicardium (see values in Table 2), assuming that it is consistent throughout the cardiac wall. Extension to the heterogeneous case can be readily incorporated into the present framework.

### Stress-assisted conduction

The mechanoelectrical feedback (the process where the current mechanical state of the deforming solid modifies both the excitability and electrical conduction of the tissue) is here introduced in the conductivity tensor, through a direct dependence on the Kirchhoff stress (which constitutes one of the main novelties in our approach, stemming as a generalisation of the anisotropy induced by stress proposed in Cherubini et al. (2017) and later used for simplified 2D cardiac electromechanics in Loppini et al. (2018)). In addition, due to the Piola transformation (yielding a transformation of the diffusion tensor using the deformation gradients), the conductivity tensor also depends nonlinearly on the deformation gradient (actually, the term $J\mathbf {C}^{-1}$ constitutes a strain-based modification of tissue conductivity, also referred to as geometric feedback in Franzone et al. (2016))

2.13 $$\begin{aligned} \mathbf {D}(v,\mathbf {F},\varvec{\varPi }) =&[D_0 + D_1 v]J\mathbf {C}^{-1} + D_0/2J {\varvec{f}_0}\otimes {\varvec{f}_0} \\&\qquad + D_2 J\mathbf {F}^{-1} \varvec{\varPi }\mathbf {F}^{\mathtt{-t}}, \end{aligned}$$

where the nonlinear conductivity (self-diffusion depending on *v*) accounts for porous media electrophysiology following the development in Hurtado et al. (2016), but appropriately modified to incorporate information about preferred directions of diffusivity according to the microstructure of the tissue (encoded in the second term defining $\mathbf {D}$). The parameter $D_0$ signifies the usual diffusion for isotropic materials, whereas $D_1$ and $D_2$ represent the intensity of the porous media electrophysiology and of the stress-assisted diffusion, respectively. An additional term in the nonlinear self-diffusion (e.g. $D_3v^2$, as in Gizzi et al. (2017), Ruiz-Baier et al. (2019)) eventually leads to very slight modifications in conduction patterns, and we have therefore decided not to include it. Tuning $D_1$ is sufficient to, if needed, calibrate the speed and action potential duration at the depolarisation plateau phase.

It is useful to point out that both the nonlinear self-diffusion term and the SAD argument derive from rigorous thermodynamical principles, formulated under specific assumptions for porous materials. In particular, nonlinear self-diffusion is naturally related to the transport of chemicals within porous media, while classical models of stress-assisted diffusion for general materials (Aifantis 1980) also consider the transport of diffusing species within solids exhibiting finite strains. For the specific case of cardiac tissue, both approaches are justified by the multiple scales involved in the transport of ions and generation and propagation of action potential within the cell and across different cells (Lenarda et al. 2018). In particular, we can mention the role of intercalated discs and gap junctions between communicating cells or the presence of the ephathic couplings in the extracellular space (Ly and Weinberg 2018), as well as micro-invaginations on the cell membrane known as microtubules and microdomains (Miragoli et al. 2016). All of these emerging effects contribute to the macroscopic nonlinearities and additional anisotropies considered in the diffusion tensor herein and which could be further analysed through a consistent multiscale homogenisation study, as well as validated using an experimental dataset.

It is important to remark that the solvability of the monodomain equations (2.11a)–(2.11b) depends on the properties of $\mathbf {D}$. In particular, the stress-assisted diffusion tensor needs to remain symmetric and uniformly elliptic, which is a non-trivial condition, given the dependence on stress and on voltage. A thorough sensitivity analysis (but for a simpler dependence on stress) can be found in Cherubini et al. (2017). Here, we perform a much lighter calibration, as mentioned later in Sect. 4. Comparisons between the effects of SAD and the more conventional mechanoelectrical feedback through stretch-activated currents have been reported in Loppini et al. (2018).

### Activation and excitation–contraction coupling

When using the active stress approach, we will adopt a simple description where the active tension is generated by ionic quantities (calcium) as well as by local fibre stretch. That is, we propose a regularised active tension model of the form

2.14 $$\begin{aligned} \partial _t T_a = {\hat{\alpha }} \varDelta T_a + \ell (T_a,\vec {r}, I_{4,f}) \qquad \text {in } \varOmega \times (0,t_{\mathrm {final}}], \end{aligned}$$

with $\hat{\alpha } = \alpha _1 D_0$, and $\ell (T_a,\vec {r}, I_{4,f}) = T_a - \alpha _2 r_3 + \alpha _3 I_{4,f}$, where $\alpha _1,\alpha _2,\alpha _3 = 0.1\alpha _2$ are positive model constants. As calcium concentration is not readily available in the phenomenological cellular model we are employing, we use $r_3$ as a proxy for intracellular calcium (Bueno-Orovio et al. 2008). In addition, a linear dependence on the calcium proxy and on the local stretch is sufficient in our setting to qualitatively capture the dynamics of active tension.

On the other hand, in the framework of active strain, a constitutive equation for the activation functions $\gamma _i$ in terms of the microscopic cell shortening $\xi$ is expressed as follows (see, for example, Barbarotta et al. 2018):

2.15 $$\begin{aligned} \gamma _f(\xi )&= \xi , \quad \gamma _s(\xi ) = (1+\xi )^{-1}(1+K_0\xi )^{-1}-1, \\ \gamma _n(\xi )&= K_0\xi , \end{aligned}$$

and the specific relation between the myocyte shortening $\xi$ and the dynamics of slow ionic quantities (in the context of our phenomenological model, only $\vec {r}$) is made precise using the law

2.16 $$\begin{aligned} \frac{d\xi }{dt} =\hat{\ell }(\xi ,\vec {r}) \qquad \text {in } \varOmega \times (0,t_{\mathrm {final}}], \end{aligned}$$

which does not require an explicit dependence on local fibre stretch, as the sliding of myofilaments is driving the dynamics of the functions $\gamma _i$. We employ the nonlinear reaction term $\hat{\ell }(\xi ,\vec {r})= K_1 (1+r_3)^{-1} + K_2 \xi$, and we make the distinction that $\ell$ and ${\hat{\ell }}$ characterise the evolution of the activation in the approaches of active stress and active strain, respectively.

## Numerical method and implementation

### Mixed-primal weak form

Restricting to the case of an active strain model with Robin conditions (2.10c) on the whole boundary for the mechanical layer (that is $\partial \varOmega _R = \partial \varOmega$) and the boundary and initial conditions (2.12a)–(2.12b) for the electrical layer, we proceed to take the inner product of the differential equations (2.8a), (2.8b), (2.9), (2.11), (2.16) with adequate test functions, and to integrate by parts whenever appropriate. We then arrive at the following weak form of the problem: for $t>0$, find $(\varvec{\varPi },\varvec{u},p)\in \mathbb {L}^2_{\text {sym}}(\varOmega )\times \mathbf {H}^1(\varOmega ) \times \mathrm {L}^2(\varOmega )$ as well as $(v,\vec {r},\xi )\in \mathrm {H}^1(\varOmega )\times \mathrm {L}^2(\varOmega )^3\times \mathrm {L}^2(\varOmega )$ such that

3.1 $$\begin{aligned}&\int _\varOmega [\varvec{\varPi }- \mathcal {G}+ pJ\mathbf {I}] : \varvec{\tau }= 0 \qquad \forall \varvec{\tau }\in \mathbb {L}^2_{\text {sym}}(\varOmega ), \\&\int _{\varOmega } \rho \partial _{tt}\varvec{u}\cdot \varvec{v}+\int _\varOmega \varvec{\varPi }\mathbf {F}^{\mathtt{-t}} : \nabla \varvec{v} \\&\quad + \int _{\partial \varOmega } \eta \mathbf {F}^{-\mathtt t} \varvec{u}\cdot \varvec{v}= \int _\varOmega \rho _0 \varvec{b}\cdot \varvec{v}\qquad \forall \varvec{v}\in \mathbf {H}^1(\varOmega ), \\&\int _\varOmega [J - 1 ] q = 0 \qquad \forall q\in \mathrm {L}^2(\varOmega ), \\&\int _\varOmega {\partial _t v}\, w + \int _\varOmega \mathbf {D}(v,\mathbf {F},\varvec{\varPi })\, \nabla v \cdot \nabla w \\&\quad - \int _\varOmega \bigl [ g(v,\vec {r}) + I_{\mathrm{ext}}\bigr ] w=0 \qquad \forall w \in \mathrm {H}^1(\varOmega ), \\&\int _\varOmega \bigl ({\partial _t \vec {r}} \cdot \vec {s} + {\partial _t \xi }\, \varphi \bigr ) - \int _\varOmega \bigl ( \vec {m}(v,\vec {r}) \cdot \vec {s} + {\hat{\ell }}(\xi ,\vec {r}) \varphi \bigr ) = 0 \\&\quad \qquad \forall (\vec {s},\varphi )\in \mathrm {L}^2(\varOmega )^3\times L^2(\varOmega ), \end{aligned}$$

where $\mathbb {L}^2_{\text {sym}}(\varOmega ) := \{ \varvec{\tau }\in \mathbb {L}^2(\varOmega ): \varvec{\tau }= \varvec{\tau }^\mathtt{t}\}$, and where the case for an active stress formulation necessitating an active tension model is addressed similarly (however, the regularity of $T_a(t)$ is then $\mathrm {H}^1(\varOmega )$). Theoretical aspects regarding the coupling of elasticity and stress-assisted diffusion problems have been recently addressed in the context of mixed-primal and mixed-mixed formulations in Gatica et al. (2018), but only for the case of simplified linear three-field elasticity and steady diffusion.

The spatial discretisation will follow a mixed-primal Galerkin approach based on the weak formulation (3.1). Details on the finite-dimensional spaces and linearisation procedure are laid out in Appendix 2.

The motivation for using three-field elasticity formulations is the need to produce robust solutions with balanced convergence orders for all variables. In addition, these methods are robust in the incompressible regime; they are not subject to volumetric locking (Lamichhane and Stephan 2012); and most importantly, they provide direct approximation of variables of interest, albeit at a higher computational cost. Another advantage of using the Kirchhoff stress is that this tensor is symmetric, and, for simpler material laws, is a polynomial function of the displacements (whereas first and second Piola-Kirchhoff stresses are rational functions of displacement) (Chavan et al. 2007). Solving in terms of stresses proves particularly useful, as this variable participates actively in the electromechanical coupling through the stress-assisted diffusion. Moreover, for the lowest-order method characterised by $l=0$, the matrix system associated with (B.1) has fewer unknowns than the discretisation that uses piecewise quadratic and continuous displacement approximations and piecewise linear and discontinuous pressure approximations [and which is a popular locking-free scheme for hyperelasticity in the displacement–pressure formulation, utilised for stress-assisted diffusion problems in the recent work (Loppini et al. 2018)]. The importance of casting the equations of motion in terms of the coupling variables has been already emphasised in Ruiz-Baier (2015) in the context of cardiac electromechanics, which demonstrates that the computation of output indicators of interest (such as conduction velocities) may suffer from loss of accuracy if one simply postprocesses stress or strain from discrete displacements as approximations in the geometric feedback.

### Solver structure and implementation details

According to the separation (through an outer fixed-point scheme) between electrophysiology and solid deformation solvers, nonlinear mechanics will be solved using the Newton–Raphson method stated above, and an operator splitting algorithm will separate an implicit diffusion solution (where another Newton iteration handles the nonlinear self-diffusion) from an explicit reaction step for the kinetic equations, turning the overall solver into a semi-implicit method. Such a strategy is feasible since the Jacobians associated with the reaction and excitation–contraction models do not possess highly varying eigenvalues (otherwise one would need to include these terms in the Newton iteration). Updating and storing of the internal variables $\xi$ and $\vec {r}$ will be done locally at the quadrature points. We solve the linear systems arising at each Newton iterate by the Krylov iterative method GMRES, preconditioned with an incomplete LU(0) factorisation (except for the linear systems in the convergence tests in Sect. 4.1, which will be solved with the direct method SuperLU), and the iterates are terminated once a tolerance of $10^{-6}$ (imposed on the $\ell ^\infty$ norm of the non-preconditioned residual) has been achieved. The mass matrices associated with the discretisation of the monodomain equations are assembled in a lumped manner, which reduces the amount of artificial diffusion and violation of the discrete maximum principle (Quarteroni et al. 2017). All routines have been implemented using the finite element library FEniCS (Alnæs et al. 2015).

## Computational results

**Table 1:** Test 1: Error history (errors on a sequence of successively refined grids and convergence rates) associated with the mixed finite element method (B.1) applied to a steady-state electromechanical coupling under active stress, and using different polynomial degrees $l\in \{0,1\}$

(a) Hyperelasticity variables							
DoF	*h*	$\Vert \varvec{\varPi }-\varvec{\varPi }_h\Vert _{0,\varOmega }$	rate	$\Vert \varvec{u}-\varvec{u}_h\Vert _{1,\varOmega }$	rate	$\Vert p-p_h\Vert _{0,\varOmega }$	rate
$$l=0$$							
77	0.7071	43.252	–	0.0576	–	30.161	–
253	0.3536	27.137	0.6725	0.0342	0.6345	19.030	0.6647
917	0.1768	12.535	1.1140	0.0216	0.7615	9.2110	1.0471
3493	0.0884	6.2636	1.0012	0.0118	0.8751	4.8012	0.9401
13637	0.0442	1.9169	1.1727	0.0071	0.9516	1.9631	1.3817
53893	0.0221	0.9841	0.9907	0.0042	0.9737	0.9206	0.9858
$$l=1$$							
221	0.7071	19.481	–	0.0146	–	6.0355	–
789	0.3536	7.9032	1.3034	0.0037	1.7593	1.5809	1.4581
2981	0.1768	2.6409	1.8079	0.0011	1.7809	0.4120	1.7269
11589	0.0884	0.7277	1.9033	4.11E−4	1.8065	0.1353	1.8813
45701	0.0442	0.2063	1.9182	1.09E−4	1.9330	0.0382	1.9602
181509	0.0221	0.0569	1.9466	3.12E−5	1.9522	0.0094	1.9571

(b) Electrophysiology variables							
DoF	*h*	$\Vert v-v_h\Vert _{1,\varOmega }$	rate	$\Vert r-r_h\Vert _{1,\varOmega }$	rate	$\Vert T_a-T_{a,h}\Vert _{1,\varOmega }$	rate
$$l=0$$							
77	0.7071	0.1528	–	0.1926	–	0.1623	–
253	0.3536	0.0902	0.7601	0.1069	0.8499	0.0847	0.8824
917	0.1768	0.0491	0.8769	0.0573	0.8968	0.0433	0.9673
3493	0.0884	0.0282	0.8016	0.0317	0.9536	0.0218	0.9896
13637	0.0442	0.0153	0.9304	0.0172	0.9612	0.0121	0.9446
53893	0.0221	0.0084	0.9587	0.0091	0.9843	0.0067	0.9562
$$l=1$$							
221	0.7071	0.0329	–	0.0583	–	0.0469	–
789	0.3536	0.0102	1.5043	0.0152	1.7317	0.0133	1.6300
2981	0.1768	0.0029	1.7608	0.0039	1.8809	0.0035	1.7095
11589	0.0884	8.03E−4	1.7849	0.0010	1.9021	9.25E−4	1.8822
45701	0.0442	2.31E−4	1.8964	2.70e−4	1.8966	2.41E−4	1.8907
181509	0.0221	6.11E−5	1.9598	7.05e−5	1.9604	6.86E−5	1.9649

### Mesh convergence

We begin with the numerical validation of our mixed-primal method on a problem slightly simpler than (2.8), (2.11), (2.14), but that still retains the main ingredients of the model. These include orthotropic active mechanics, nonlinear reaction–diffusion with stress-assisted diffusion, and a nonlinear excitation–contraction coupling.

A convergence test is generated by computing errors between smooth exact solutions and approximate solutions using the first-order and the second-order methods discussed in Sect. 3. Let us consider the following closed-form solutions to a steady-state counterpart of the variational form (3.1) for the electromechanics equations, also assuming the absence of viscoelastic effects, and defined on the domain $\varOmega =(0,1)^2$ with the fibres/sheetlets defined as ${\varvec{f}_0}=(0,1)^\mathtt{t},{\varvec{s}_0}= (-1,0)^\mathtt{t}$

$$\begin{aligned} \varvec{u}(x,y)&= 0.1\begin{pmatrix} \sin (\pi x)\cos (\pi y)\\ \cos (\pi x)\sin (\pi y)\end{pmatrix},\\ p(x,y)&= 0.1\sin (\pi x)\sin (\pi y),\\ v(x,y)&= 1+0.1\cos (\pi x)\cos (\pi y),\\ r(x,y)&= 0.1\cos (\pi x)\sin (\pi y)\sin (\pi x),\\ T_a(x,y)&= 1+0.1\cos (\pi x)\sin (\pi y){.} \end{aligned}$$

Then, the Kirchhoff stress $\varvec{\varPi }$ and forcing terms (volumetric load, an additional external stimulus, and the active tension source) are computed from these smooth solutions, the balance equations, relations (2.2), (2.3), (2.13), and using the following simplified constitutive equations

$$\begin{aligned} m(v,r) = v-r^2, \ g(v,r) = (v-1)vr, \ \ell (T_a,r) = - T_a + r. \end{aligned}$$

Note also that the incompressibility constraint for this test is $J=J_{ex}$, where $J_{ex}$ is computed from the exact displacement. Here, we also prescribe Dirichlet boundary conditions for displacements, transmembrane potential, and active tension (incorporated in the discrete trial spaces). Errors due to fixed-point iterations are avoided by taking a full monolithic coupling and computing solutions using Newton–Raphson iterations with an exact Jacobian. On a sequence of six uniformly refined meshes, we proceed to compute errors between the exact and approximate solutions computed with methods using $l=0$ and $l=1$. Kirchhoff stress and pressure errors are measured in the $L^2-$norm, whereas for the remaining variables, the errors are measured in the $H^1-$norm. The obtained error history is reported in Table 1, where we observe an asymptotic $O(h^{l+1})$ decay of the error for each field variable. This behaviour corresponds to the optimal convergence according to the interpolation properties of the employed finite element subspaces (Chavan et al. 2007).

**Table 2:** Model parameters for the electro-viscoelastic model (2.8), (2.11), (2.16), (2.14). Values are taken from Cherubini et al. (2017), Gao et al. (2015), Rossi et al. (2014), Bueno-Orovio et al. (2008), and the transmembrane potential *v* is dimensionless

Viscoelasticity constants							
$a= 0.236$	[N/cm$^2$]	$a_{f}=1.160$	[N/cm$^2$]	$a_{s}= 3.724$	[N/cm$^2$]	$a_{fs}= 4.010$	[N/cm$^2$]
$b= 10.81$	[–]	$b_{f}= 14.15$	[–]	$b_{s}= 5.165$	[–]	$b_{fs}= 11.60$	[–]
$p_0=0.1$	[N/cm$^2$]	$\beta = 10$	[ms]	$\delta = 22.6$	[N/cm$^2$ ms]	$\zeta _{\text {stab}}= 0.25$	[–]
$\eta _a= 0.001$	[N/cm$^2$]	$\eta _b= 0.01$	[N/cm$^2$]	$\kappa _{sn}=0.6$	[–]	$\kappa _{nn}= 0.03$	[–]
$\rho _0= 0.001$	[N/cm$^2$]						

Electrophysiology constants							
$v_0= 0$	[–]	$v_v=1.55$	[–]	$v_2^-=0.03$	[–]	$v_{so}=0.65$	[–]
$v_3= 0.908$	[–]	$\theta _1= 0.3$	[–]	$\theta _1^-=0.006$	[–]	$\theta _{o}= 0.006$	[–]
$\theta _2=0.13$	[–]	$k_2^-=65$	[–]	$k_3=2.099$	[–]	$k_{so}=2.045$	[–]
${r^*_{2,\infty }}=0.94$	[–]	$\tau _{2,\infty }= 0.07$	[–]	$\tau _{1,1}^-=60$	[–]	$\tau ^-_{1,2}=1150$	[–]
$\tau ^-_{2,1}= 60$	[–]	$\tau ^-_{2,2}=15$	[–]	${\tau _{fi}}=0.11$	[–]	${\tau _{o,1}}=30.02$	[–]
${\tau _{o,2}}=0.996$	[–]	$\tau _{so,1}=2.046$	[–]	$\tau _{so,2}=0.65$	[–]	$\tau _{3,1}= 2.734$	[–]
$\tau _{3,2}=16$	[–]	${\tau _{si}}=1.888$	[–]	$\tau ^+_{1}=1.451$	[–]	$\tau ^+_{2}=200$	[–]
$$\chi = 1$$	[–]						

Activation and excitation–contraction coupling constants							
$D_0= 1.171$	[cm$^2$/s]	$D_1= 0.9$	[cm$^2$/(s mV)]	$D_2= 0.01$	[cm$^2$/(s Pa)]	$K_0= 5$	[–]
$K_1= -0.015$	[–]	$K_2= -0.15$	[–]	$\alpha _1= 10$	[–]	$\alpha _2= 0.5$	[–]

### Parameter calibration

**Fig. 2:**
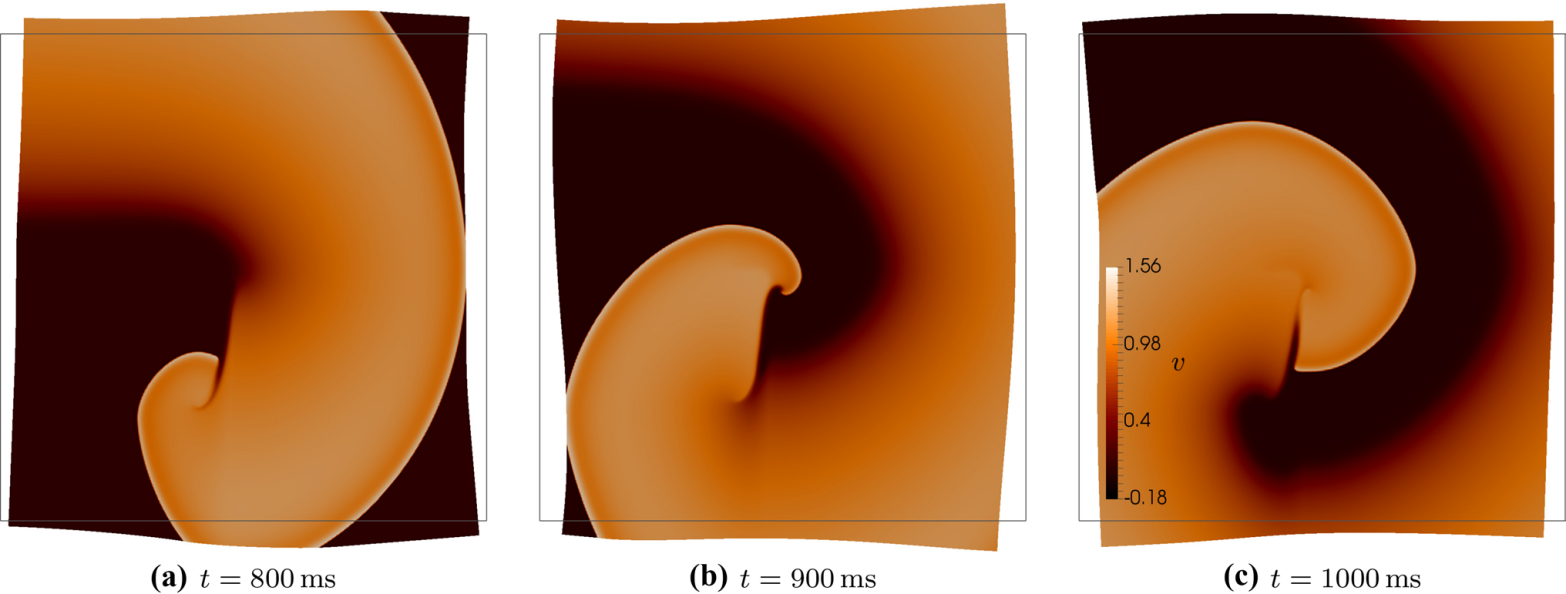
Evolution of voltage after S2 stimulus, of S1-S2 protocol, showing formation of a reentrant spiral wave on the deforming viscoelastic tissue, computed using the active stress approach

For the following 2D simulations, we will initially consider tissue slabs of $50 \times 50$ mm$^2$, and set fibre and sheetlet directions simply as ${\varvec{f}_0}= (1,0)^\mathtt{t}$, ${\varvec{s}_0}= (0,-1)^\mathtt{t}$. The initiation, maintenance, prevention, and treatment of so-called reentrant waves are major focus of the current research due to their implication in atrial and ventricular fibrillations (Franzone et al. 2014). We are thus interested in investigating the formation of spiral reentrant waves in our model setup, following the S1-S2 stimulation protocol. We first excite the tissue with a symmetric stimulus labelled S1. An asymmetric stimulus labelled S2 is then applied during the vulnerable window near the end of the refractory period, when some of the tissue has recovered excitability, but depolarisation is still blocked elsewhere. This causes unbalanced excitation, which can lead to the formation of a spiral wave. We will define the spiral front as the edge of the spiral wave, where the excitation front meets the repolarisation waveback of the action potential. In our simulations, both waves have non-dimensional amplitude 3 and duration 3 ms. The S1 stimulus is a planar wave created by exciting the entire left edge of the tissue, and the S2 stimulus is a square wave created by exciting the bottom left quadrant at *t* = 330, ms. In 3D, the same general protocol can be used, but the S1 stimulus excites the entire bottom section (below some value of the $z-$coordinate) while the S2 stimulus excites the bottom left octant at $t = 335$ ms. Here, we use the active stress approach, and the boundary conditions for the visco-elastodynamic equations correspond to (2.10c). The formation and evolution of the spiral wave on a deforming domain can be seen in Fig. 2. The spiral is initiated by the diffusion of voltage and transport of ionic entities from the S2 stimulus into the leftmost section of the tissue, which has recovered enough excitability after S1. The wave then spreads outwards in all directions, occupying the entire tissue except for the region that was just excited by the S2 wave.

**Fig. 3:**
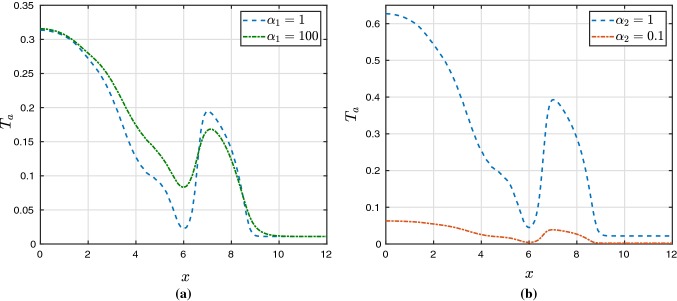
Profiles of $T_a$ taken across a smaller slab of tissue at $y=6\,\text {mm}$ and $t=432\,\text {ms}$. These plots evaluate the effect of $\alpha _1$ and $\alpha _2$

Next, since we are using the active stress formulation in this case, we proceed to evaluate $\alpha _1, \alpha _2$, the parameters governing active tension in (2.14), and $\eta$, the stiffness parameter from (2.10c). We conduct a simple sensitivity analysis by increasing or decreasing either $\alpha _1, \alpha _2$, or $\eta$ by one order of magnitude, holding the others constant at their reference values ($\alpha _1 = 10$, $\alpha _2 = 0.5$, and $\eta = \eta _a = 0.001$N/cm$^2$, as listed in Table 2). This simple analysis therefore does not test for compounding or interaction effects. We also consider a smaller slab of size $12 \times 12$ mm$^2$. The parameter $\alpha _1$ contributes to producing smoother active tension profiles, while $\alpha _2$ controls the range of their magnitude. These effects are visible in Fig. 3. We found that larger values of $\alpha _1$ produced smoother gradients in pressure and stress, while larger values of $\alpha _2$ produced, in average, higher magnitude displacement, Kirchhoff stress, and pressure, as well as some more subtle changes in ionic quantities. Parameter $\eta$ determines the stiffness of the springs supporting the tissue, and so decreasing $\eta$ resulted in an increase in the maximum values of magnitude of displacement, stress, and pressure, as expected. However, these differences were minimal, even across the three orders of magnitude tested ($\eta = 1$E−4 to $\eta = 0.01$). The effects on ionic entities were even smaller, for both the hyperelastic and viscoelastic cases, and therefore, plots are not shown.

Computational experiments reveal a window of values of $D_2$ for which our method converges. In the 2D hyperelastic case, we found that the upper bound for $D_2$ is approximately $D_2 = 2.1$E−2 cm$^2/$(s Pa), with the linear solver failing to converge for larger values. In these simulations, the Kirchhoff stress achieved an $L^2-$norm of between 0.006 and 0.6. In turn, the viscoelastic case was able to accept slightly larger values of $D_2$, up to $D_2 = 2.2$E−2 cm$^2/$(s Pa), with the $L^2-$norm of stress falling between 0.001 and 0.5. A possible explanation is the loss of coercivity or monotonicity in the stress-assisted diffusion coupling, as explored in Cherubini et al. (2017).

The numerical method used for these tests is characterised by the time step, mesh size, polynomial degree, and stabilisation constant $\varDelta t= 0.01$ ms, $h=0.3534$ mm, $\ell = 0$, $\zeta _{\mathrm {stab}} = 2.5$, respectively.

### Locking-free property

We next proceed to assess the performance of the proposed mixed formulation for the mechanical problem. In this example, we solve only for (2.8) without the acceleration term (otherwise present in all other simulations), using the active stress approach with a fixed value for the active tension and without the contribution from the viscous stress (2.6). We proceed to compare the deformation achieved by the mixed formulation with that of an asymptotic solution and the approximate solution generated by a more standard pressure-displacement finite element formulation. We consider different stabilisation parameter values and mesh refinements.

**Fig. 4:**
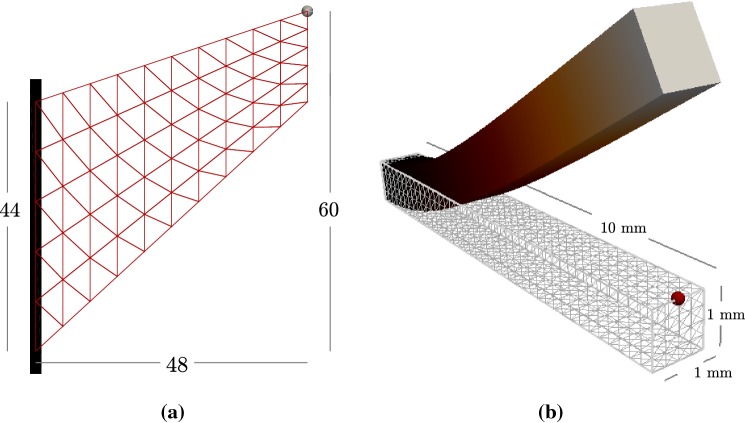
Domain sketches and sample meshes for the deflection of Cook’s membrane for an Holzapfel–Ogden material with constant active stress (**a**) and deflection of a 3D beam for a Guccione–Costa–McCulloch material with the active stress component set to zero (**b**)

**Fig. 5:**
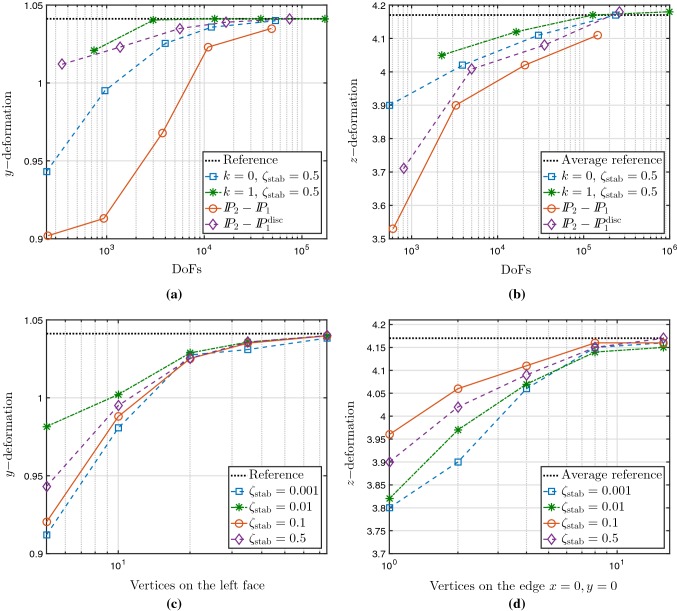
Convergence of the deflection of Cook’s membrane for an Holzapfel–Ogden material with constant active stress (**a**, **c**) and deflection of a 3D beam for a passive Guccione–Costa–McCulloch material (**b**, **d**). Maximal vertical deflection with respect to the mesh resolution for different numerical schemes (**a**, **b**), and different values of the stabilisation constant (**c**, **d**)

We perform two sets of computations. First, we undertake Cook’s membrane benchmark test for a fully incompressible Holzapfel–Ogden material (as was similarly done for nearly incompressible Saint Venant–Kirchhoff and neo-Hookean solids in (Chavan et al. 2007, Test II)), where we set an active tension of $T_a = 0.07$. This test involves applying an upward in-plane shear load $\varvec{t}=(0,100)^\mathtt{t}$ to the right edge of a tapered panel with a clamped left edge, and measuring the vertical deformation of the upper right vertex. The domain is defined as the convex hull of the set $\{(0,0),(48,44),(48,60),(0,44)\}$ (see the sketch in Fig. 4a), and the fibre and sheetlet fields are ${\varvec{f}_0}=(1,0)^\mathtt{t}$ and ${\varvec{s}_0}= (0,-1)^\mathtt{t}$, respectively. Secondly, we consider a 3D system suggested in (Land et al. 2015, Test I) as a simple benchmark for passive cardiac mechanics, and therefore, we set $T_a=0$. The problem consists in computing the deformation of a point at the right end of a beam defined by the domain $\varOmega = (0,10)\times (0,1)\times (0,1)$  mm (see Fig. 4b), where the fibre direction is ${\varvec{f}_0}=(1,0,0)^\mathtt{t}$. Instead of (2.1), the material is characterised by the transversally isotropic strain energy density proposed by Guccione et al. (1995) [which is the material law used in the benchmark test from Land et al. (2015)]: $\varPsi _{\text {pas}} = a/2 (e^Q-1)$, with $Q = b_f E_{ff}^2 + b_t (E_{ss}^2+ E_{nn}^2 + E_{sn}^2+E_{ns}^2) + b_{fs} (E_{fs}^2+ E_{sf}^2 + E_{fn}^2+E_{nf}^2)$, where $a=2$ kPa, $b_f=8$, $b_t=2$, $b_{fs}=4$, and the $E_{ij}$ denote entries of the Green–Lagrange strain tensor $\mathbf {E}$, rotated with respect to a local coordinate system aligned with ${\varvec{f}_0},{\varvec{s}_0},{\varvec{n}_0}$. The beam is clamped at the face $x=0$, a pressure of $p_N=0.004$ kPa is imposed on the bottom face $z=0$, and the remainder of the boundary is considered with traction-free conditions. According to (2.10b), the pressure boundary condition changes with the deformed surface orientation, and its magnitude scales with the deformed area.

The outcome of these tests in Fig. 5a, b shows a rapid convergence of our first- and second-order methods, while the computations using a pressure-displacement formulation and the Taylor–Hood finite elements (the well-known $\mathbb {P}_2-\mathbb {P}_1$ pair of continuous and piecewise quadratic approximations of displacements and continuous and piecewise linear approximations for pressure) display a somewhat slower convergence to the asymptotic deflection of the membrane. Using discontinuous pressures (the $\mathbb {P}_2-\mathbb {P}_1^{\text {disc}}$ pair) rectifies the convergence, but at a higher computational cost. Quite similar results were obtained for the beam (where the reference value is the average of the reported simulations from the study in Land et al. (2015)). Moreover, Fig. 5c, d shows the vertical deflections as a function of the number of vertices discretising the left side of the membrane and of the small edge of the beam, respectively. They indicate that the obtained results are consistent for varying values of the stabilisation parameter, $\zeta _{\text {stab}}$, and the observed behaviour also confirms that our method is locking-free.

### Stress-assisted diffusion and conduction velocity

In addition to determining a suitable parameter range for $D_2$ that ensures solvability of the discrete monodomain equations, we also investigated the effect of $D_2$ on the tissue’s response to spiral wave dynamics. As in the second part of Sect. 4.2, this time the domain is a square slab of width $12\,\mathrm{mm}$ aligned with the canonical axes. We employ the active stress approach and use Robin boundary conditions for the viscoelasticity problem. The fibres assume the fixed direction ${\varvec{f}_0}=(1,0)^\mathtt{t}$ and the sheetlets ${\varvec{s}_0}= (0,-1)^\mathtt{t}$, and the Holzapfel–Ogden material law is considered. The mesh size is approximately $h = 0.085\,$ mm and the time step is $\varDelta t = 0.1\,$ms. We use the lowest-order finite element method and the stabilisation parameter is $\zeta _{\text {stab}} = 2.5$.

**Fig. 6:**
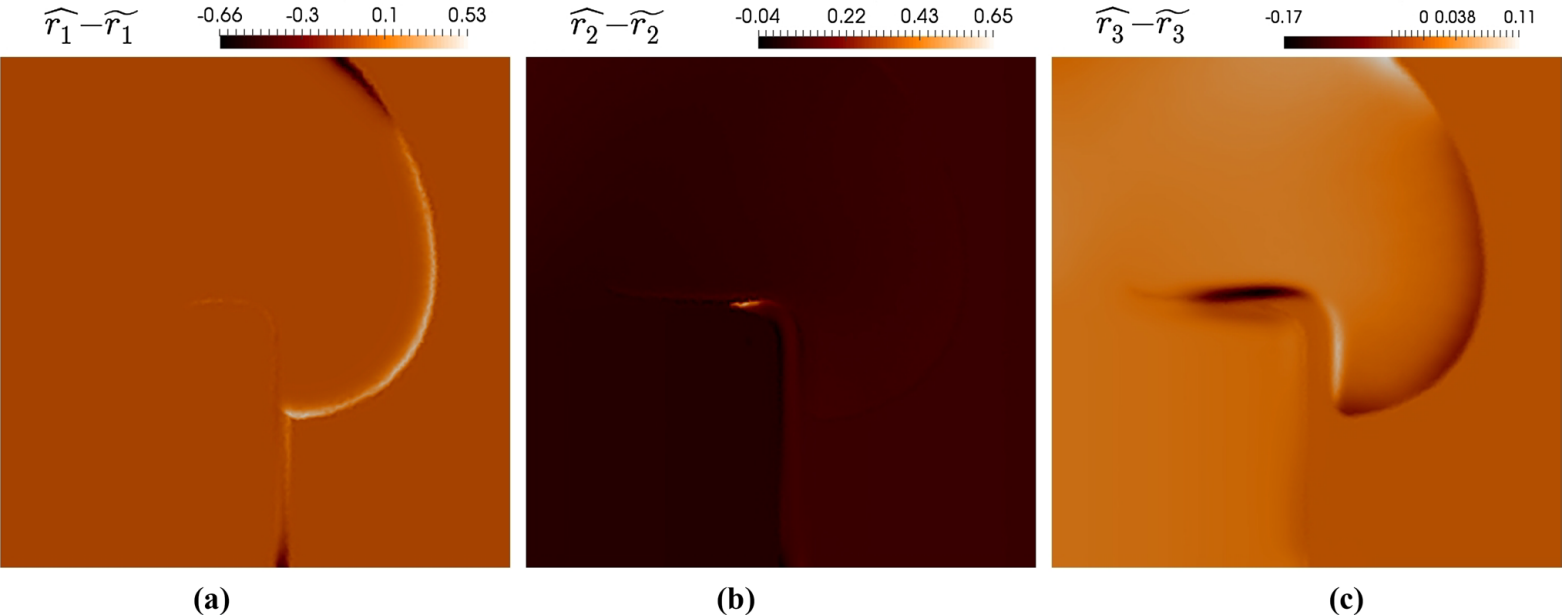
Differences in ionic quantities from varying SAD parameter $D_2$ at $t=444\,\text {ms}$. Quantities $\widehat{r_i}$ indicate the profiles with $D_2=$7.5E−3, and $\widetilde{r_i}$ the profiles associated with $D_2=$1.0E−5 (dimensions are as in Table 2)

**Fig. 7:**
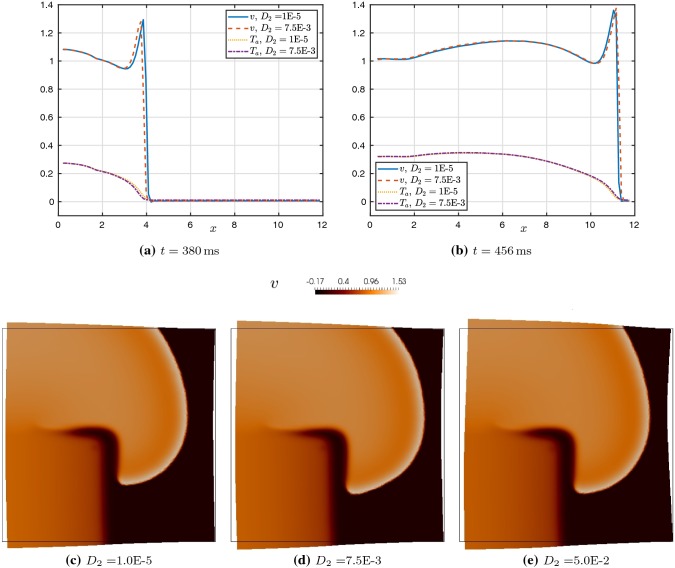
**a**, **b** Propagation of action potential *v* and active tension $T_a$, measured by taking the profile over a horizontal line segment crossing the upper half of the tissue at $y=7\,\text {mm}$. Comparison is provided for two different values of $D_2$. **c**, **d**, **e** Effect of $D_2$ on the potential wave at $t=444\,\text {ms}$ in the viscoelastic case

Figure 6 shows the differences in the ionic quantities between simulations with a very small contribution of SAD ($D_2 =$ 1 E−5 m$^2/$(s Pa)) and a more prominent, but still mild SAD contribution ($D_2 =$7.5E−3 cm$^2/$(s Pa)). The snapshots correspond to the time $t = 444$ ms, when the spiral tip has not yet formed. A closer inspection suggests that these contrasts were due to a difference in conduction velocity induced by SAD. In Fig.  7a, b, we see that conduction velocity was higher for larger values of $D_2$ (meaning a larger SAD contribution). When the wave first emerged, the peak action potential was more advanced for the case of reduced $D_2$, but the large $D_2$ peak eventually caught up to and surpassed it, which is a phenomenon also observed in the active tension curves. The ionic quantities followed the same trend. Indeed, an analysis similar to that which produced Fig.  7a, b revealed that the overall profiles of the ionic quantities were highly similar between the two cases compared in Fig. 6, but differed in the speed at which they are transported through the tissue.

We also remark that the effect of changing conduction velocities was not spatially consistent. For some parameter values, SAD increases conduction velocity in the fibre (horizontal) direction, but actually decreased conduction velocity in the vertical and diagonal directions. This resulted in a noteworthy effect on the growth of the spiral wave. Figure 7c–e shows a comparison of the spiral wave in the viscoelastic case for three different values of $D_2$. The upper right area of the spiral is slightly more vertical in the simulation with a larger value of $D_2$ than in the other cases, suggesting that propagation of the voltage is somewhat hindered in that direction. We also observe a slightly more pronounced deformation of the right side of the domain due to the two-way coupling between tissue motion and electrophysiology. A similar effect was seen in the hyperelastic case.

As in other studies, here we observe that conduction velocity is sensitive to spatiotemporal discretisation. In Table 3, we include the results of a simple convergence test for conduction velocity, similar to the benchmark test conducted in Ruiz-Baier et al. (2019). We calculated the horizontal propagation of the action potential using different time steps and mesh refinements. Differently to the case of nonlinear diffusion without SAD from Ruiz-Baier et al. (2019), the experiment reveals that lower resolutions produce larger conduction velocities than the physiological values. This test also confirms that with our time step and mesh resolution ($0.1\,\text {ms}$, and above 200, 000 DoF, respectively), conduction velocity is in the expected physiological range, whereas larger time steps will systematically fail to capture the dynamics of the ionic model.

**Table 3:** Convergence of conduction velocity with respect to temporal and spatial discretisation

Convergence of conduction velocity, mm/ms					
DoF	h (mm)	$\varDelta t= 0.3\,\text {ms}$	$0.1\,\text {ms}$	$0.05\,\text {ms}$	$0.01\,\text {ms}$
27038	0.3817	0.1130	0.1032	0.1015	0.0994
108576	0.1909	0.0754	0.0705	0.0654	0.0637
170919	0.1527	0.0733	0.0657	0.0632	0.0620
246456	0.1273	0.0701	0.0632	0.0601	0.0589
554960	0.0849	0.0649	0.0553	0.0551	0.0550
1204362	0.0768	0.0610	0.0552	0.0550	0.0547

**Fig. 8:**
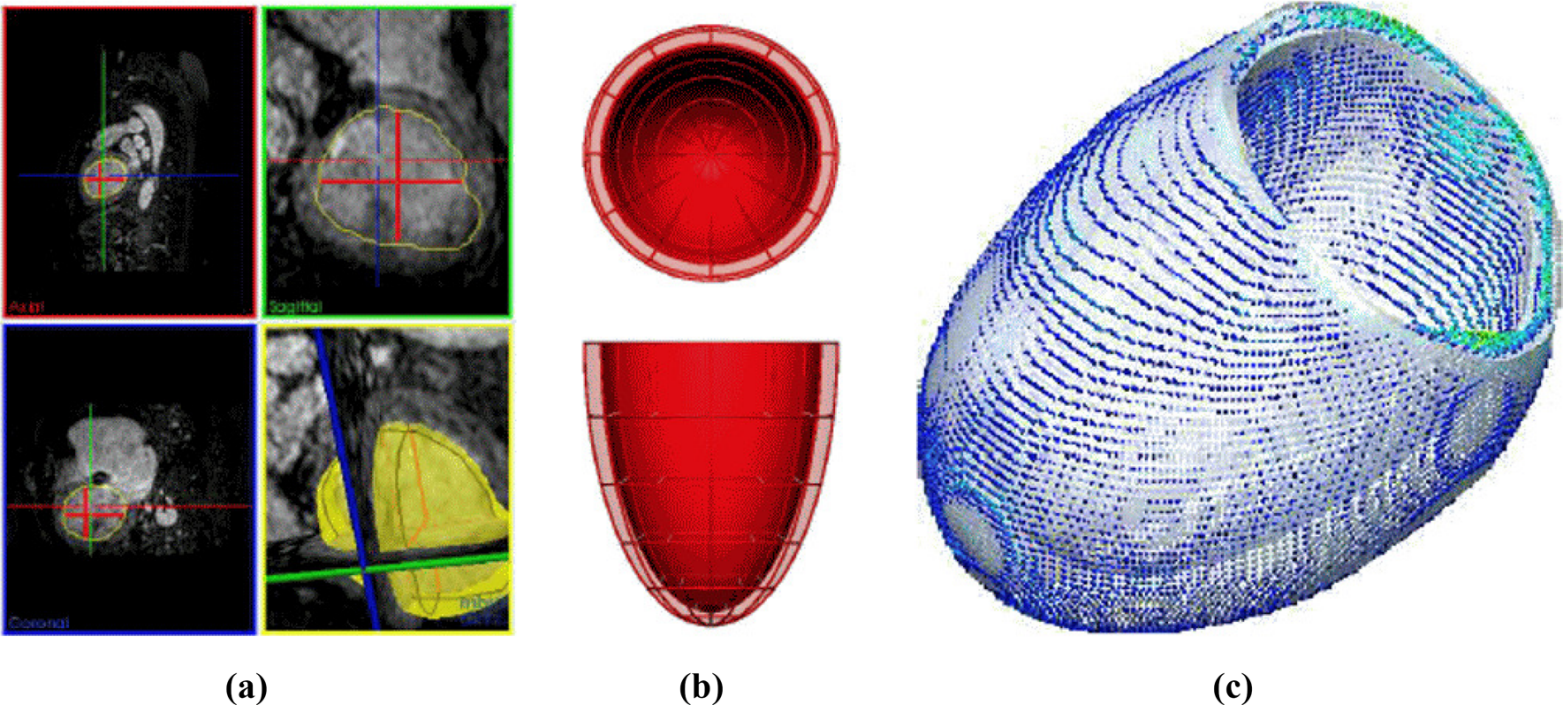
Segmentation and mesh personalisation process from Warriner et al. (2018), Lamata (2018). Semi-automatic segmentation by 3D extrapolation (yellow surface and contours) of 2D segmentation contours (red contours and projections) (**a**); surface mesh template (**b**); and resulting mesh (white surface) overlaid with the segmentation surface colour coded by the distance between them (jet colour map, from 0 mm in blue to 1 mm in red) (**c**). Used with permission

### Scroll waves on mono-ventricular geometries

For the ventricular geometries, we test both the active strain and active stress formulations. We start from patient-specific left ventricular geometries [available from Warriner et al. (2018), Lamata (2018)] and rescale them using approximately the same dimensions as the idealised ventricles studied in Ruiz-Baier et al. (2019). The segmentation process is outlined in Fig. 8. From there we define boundary labels and produce volumetric tetrahedral meshes of varying resolutions. The domain boundaries are set as sketched in Fig. 1: the basal cut corresponds to $\partial \varOmega _D$, the epicardium to $\partial \varOmega _R$, where the Robin boundary conditions (2.10c) are defined with a spatially varying stiffness

$$\begin{aligned} \eta (y) = \frac{1}{y_b-y_a}[\eta _a(y_b-y) + \eta _b(y-y_a)], \end{aligned}$$

and the endocardium to $\partial \varOmega _N$, where we set $p_N(t) = p_0\sin ^2(\pi t)$, representing the variation of endocardial pressure. The constants $y_a,y_b$ are the vertical components of the apical and basal locations, and $\eta _a<\eta _b$ denotes the stiffness sought at the apex and base, respectively (assuming that the contact of the muscle with the aortic root is more resistant to traction than the more flexible pericardial sac and surrounding organs). In addition, since fibre and sheetlet fields for mono-ventricular geometries are not usually extracted from MRI data, we generate them using a mixed-form adaptation to the Laplace–Dirichlet rule-based method proposed in Wong and Kuhl (2014), Rossi et al. (2014).

**Fig. 9:**
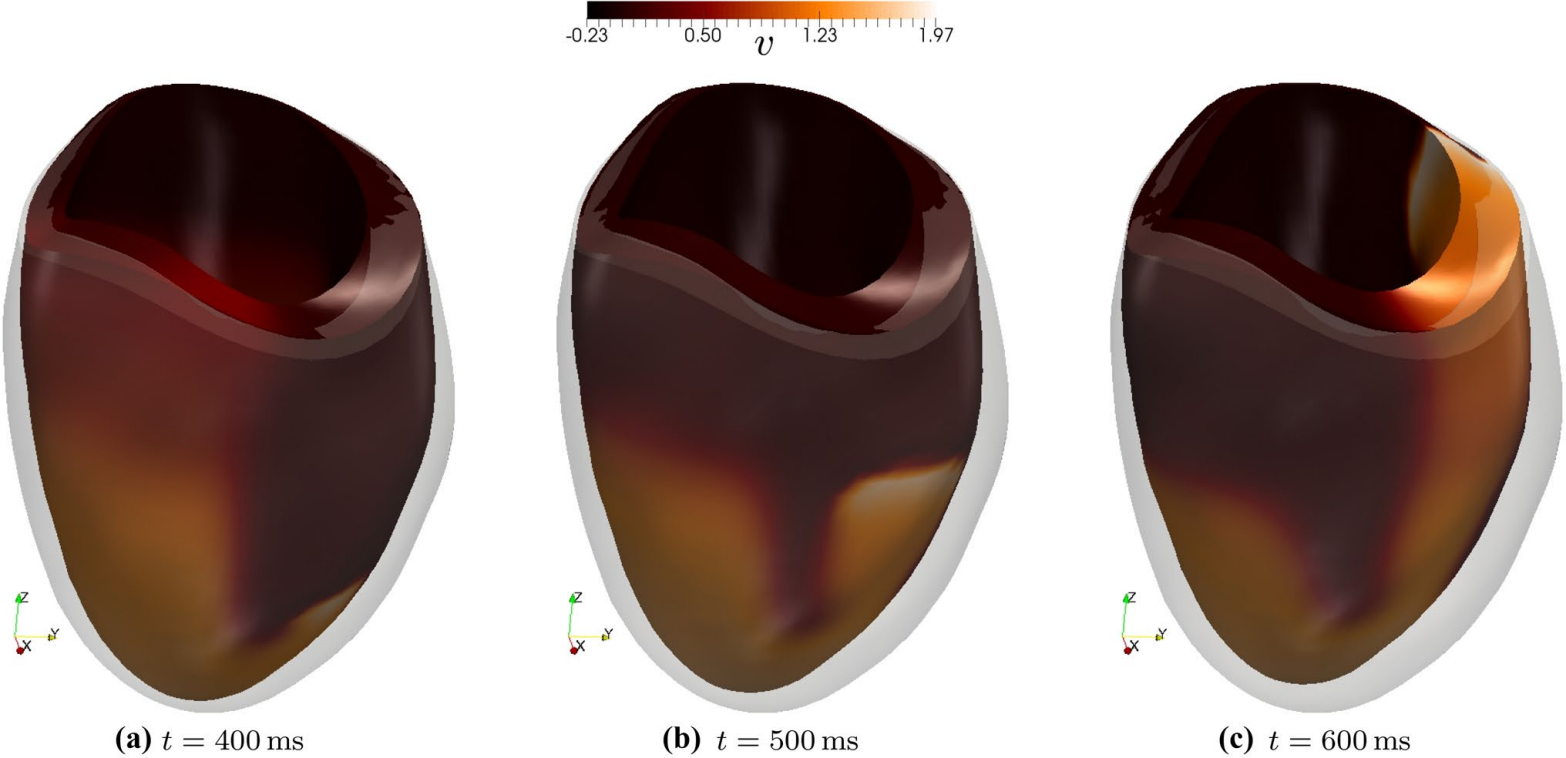
Evolution of voltage after S2 stimulus (at $t=335\,\text {ms}$), showing formation of a scroll wave on a contracting ventricle, using the active strain model. The shadow of the undeformed ventricle geometry is shown for comparison

**Fig. 10:**
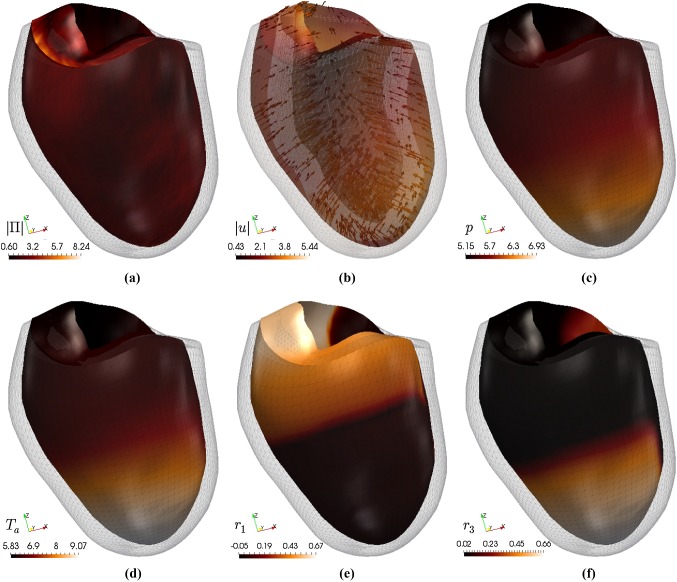
Snapshot at $t=600\,\text {ms}$ of field variables plotted on the deformed domain and less opaque undeformed mesh. Here we have also used the active strain approach

After the S2 stimulus excites a group of cells in the lower left octant at $t = 335$ ms, a spiral wave forms and sweeps around both sides of the ventricle, the two sides merging at approximately $t = 415$ ms. Simultaneously, we see contraction of the apical region in the upwards direction, complemented by torsion and thickening of the ventricle wall. Figure 9 shows the propagation of the action potential on the deforming ventricle, with the original ventricle geometry shown with reduced opacity for comparison. The S2 stimulus occurs on the apex, and the nascent scroll wave is not visible until the two arms of the wave interact. For these tests the mesh size was approximately $h = 0.24\,$ mm and the time step $\varDelta t = 0.1\,$ms. We have employed the lowest-order finite element method $l=0$ and the stabilisation parameter is taken as $\zeta _{\text {stab}} = 25$ (Fig. 10).

**Fig. 11:**
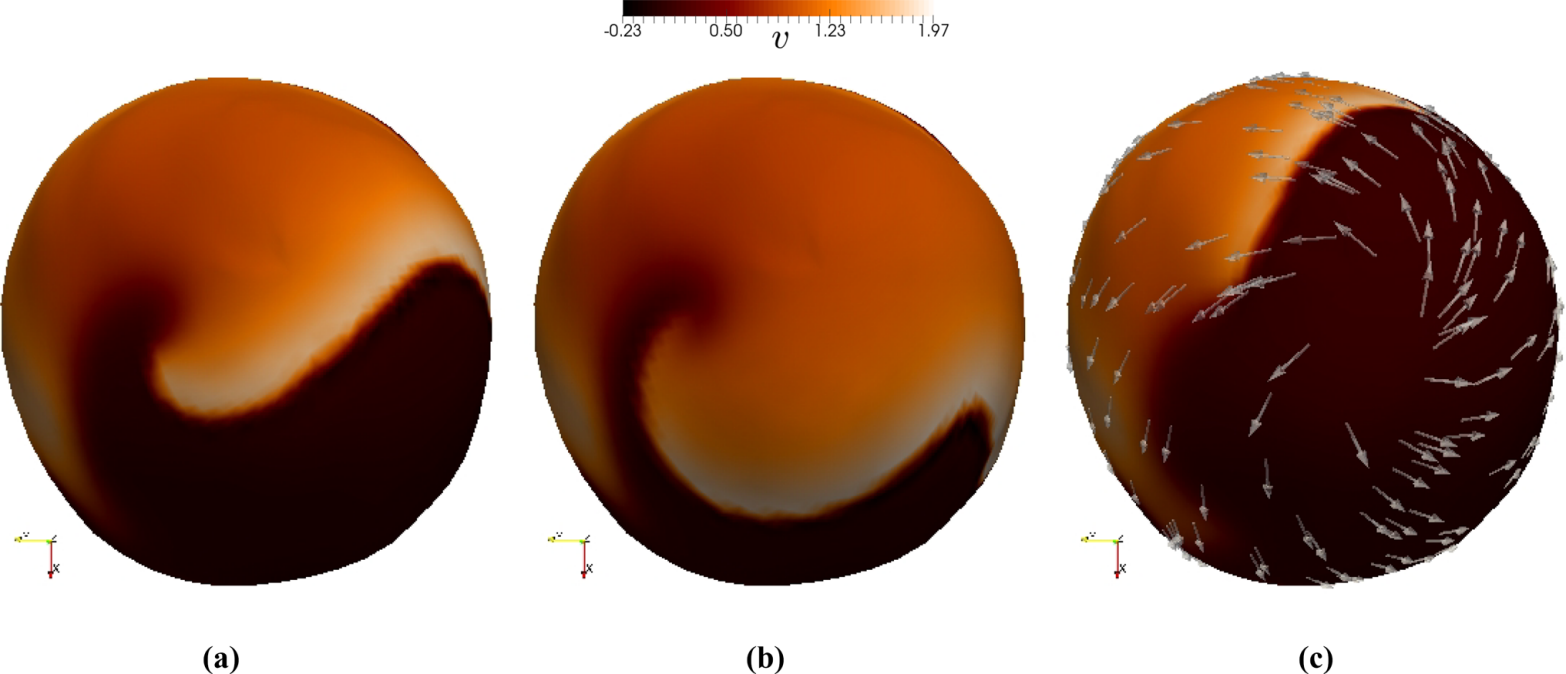
Effect of SAD on spiral wave propagation, using the active strain formulation. Panels **a**, **b** show voltage and **c** shows the difference between the SAD and non-SAD cases $v_\text {SAD}-v_\text {non-SAD}$ (which has a different scale). The action potential wave using SAD moved along the fibre direction ahead of the non-SAD case

In addition, and similarly to the 2D case, incorporating SAD impacted the propagation of the spiral wave anisotropically. In the fibre direction, SAD led to earlier advancement of the spiral. In the transverse direction, the non-SAD case advanced earlier. Figure 11 shows the difference in voltage for the two cases (along with the actual voltage profile, for reference). The effect seen in the fibre direction (indicated by the white arrows) was not seen in the other directions. For these tests, we have used the active strain formulation and we have included viscoelastic effects, as well as inertial contributions.

**Fig. 12:**
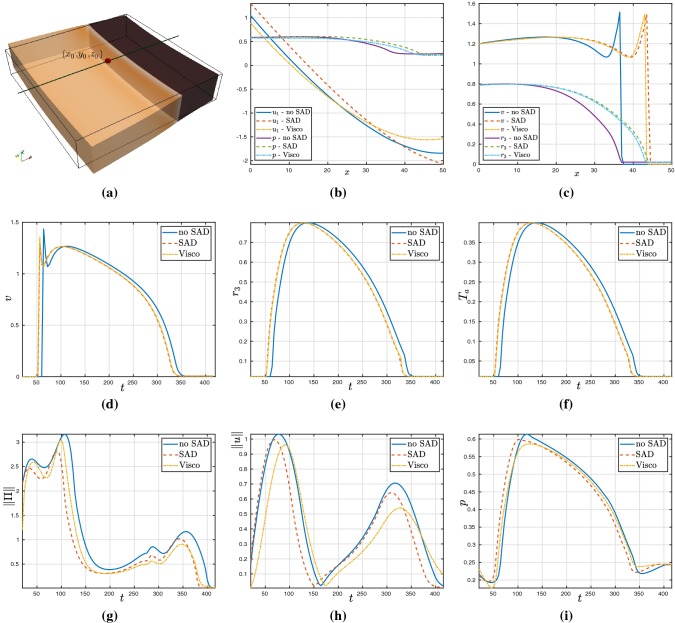
Comparison of field variables between hyperelastic and viscoelastic cases on a line parallel to the *x* axis (sketched in **a**) taken at $t=92\,\text {ms}$ (**b**, **c**); and pointwise evolution of field variables on the point $(x_0,y_0,z_0)$ (**d**–**i**) for the cases of hyperelasticity without SAD, with the baseline case of SAD but without viscous stresses, and the viscoelastic case (line, dashed, and dashed-dotted curves, respectively). For these tests, we have used the active stress formulation

### Effects due to viscoelasticity

In order to quantify the discrepancies between hyperelastic and viscoelastic effects, we conduct a series of simulations using the coupled model on a 3D slab of dimensions $50\times 50\times 10$ mm$^3$ using a tetrahedral mesh of $h = 0.25$ mm, also setting $\varDelta t = 0.1\,$ ms, $\zeta _{\text {stab}} = 25$, and ${\varvec{f}_0}=(1,0,0)^\mathtt{t}$, ${\varvec{s}_0}=(0,1,0)^\mathtt{t}$. These tests are conducted using the active stress formulation, and we consider inertial effects. We apply a S1 stimulus on the face $x=0$, and after $t= 92\,\text {ms}$, the propagation front has reached the state shown in Fig. 12a, plotted on the deformed configuration (which was computed with a full electro-viscoelastic model). The boundary conditions for the viscoelasticity are of Robin type everywhere on the boundary. At that time, in panels (b,c) we depict snapshots of the approximate solutions obtained using the hyperelastic and viscoelastic models with their baseline parameter values as reported in Table 2, and shown over a line segment crossing the tissue slab parallel to the $x-$axis. We show profiles of the mechanical entities ($x-$components of displacement and pressure), as well as potential and $r_3$. For reference, we also include the results obtained using a model without SAD contributions (that is with $D_2=0$). We note that the curves produced without SAD are substantially lagged (as expected from the choice of diffusion parameters) with respect to the two other cases that display no major discrepancies. The remaining panels in the figure show pointwise transients of the main mechanical and electrical fields measured on the point $(x_0,y_0,z_0)=(25,25,10)$. The evolution of the electric and activation fields remains very similar in all three cases; for instance, the shape of the action potential is almost not modified after adding SAD or viscous contribution and for the other fields also very subtle differences are observed. (The calcium concentration was slightly shifted to the left in the hyperelastic and viscoelastic cases.) The changes are more pronounced in the Frobenius norm of the Kirchhoff stress, the displacement magnitude, and the pressure (panels g,h,i). These computations suggest that viscous effects will result in a decreased displacement, stress, and pressure (similar conclusions were drawn in Pandolfi et al. (2017), but not in the context of models for ventricular viscoelasticity). These discrepancies, however, are qualitatively small, and this observation was robust to every parameter combination that we tested, consistent spatially and in time. The application of a viscous model also had consequences related to performance. For instance, in the tests mentioned above, the average number of Newton iterations needed to reach convergence was systematically lower in the viscous case than in the hyperelastic case. This behaviour is expected as for simple viscoelastic models the tangent problem is essentially a rescaled version of the elastic stiffness, which contributes to improving the stability of the linearised system.

**Fig. 13:**
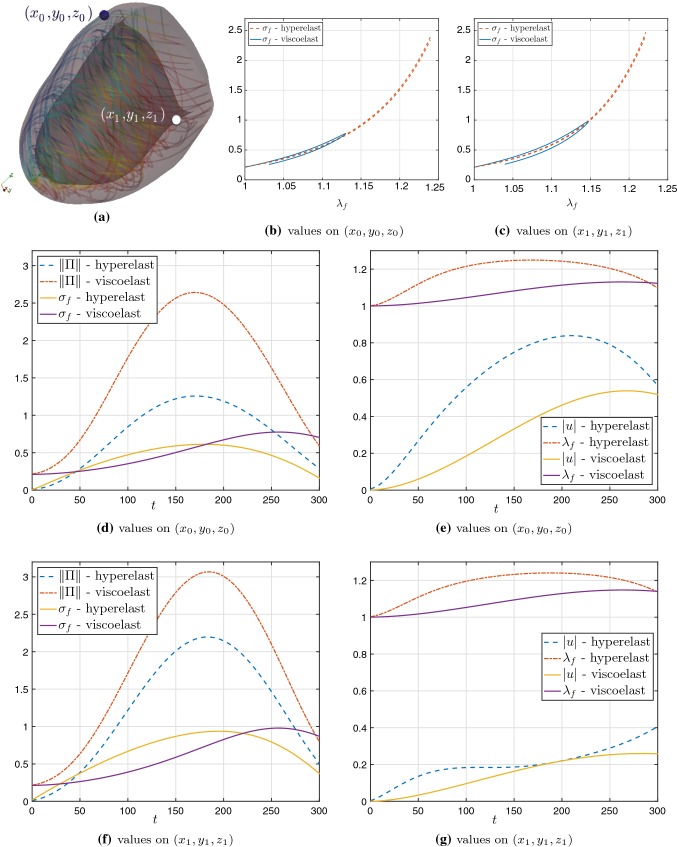
Comparison between hyperelastic and viscoelastic deformation under passive inflation. True stress in the fibre direction $\sigma _f = \mathbf {F}{\varvec{f}_0}\cdot (\varvec{\sigma }\mathbf {F}{\varvec{f}_0})$, measured according to local stretch on two points on the epicardium (**b**) and endocardium (**c**) [points indicated in panel (**a**)]. The plots in panels (**d**, **e**) show transients of mechanical outputs (Frobenius norm of the Kirchhoff stress, true stress on fibre direction, local stretch, and displacement magnitude) at the point $(x_0,y_0,z_0)$; and plots (**f**, **g**) display their counterparts in point $(x_1,y_1,z_1)$. For these tests we have used only inertial effects and passive hyperelastic or viscoelastic contributions

We next proceed to investigate the effects of changing the viscosity parameters. The parameter $\beta$ from (2.6) exerted minimal influence over the observed dynamics. Even for the five orders of magnitude tested, from $\beta = 0.1$ ms to $\beta = 10{,}000$ ms, the differences in displacement, voltage, and all other variables were of less than 0.1%. This could be because of the low rates of change of deformation that we see in our simulations. We also tested values of $\delta$ across three orders of magnitude, from $\delta = 2.26$ to $\delta = 2260$ (in units N/cm$^2\cdot$ms). As expected, increasing this quantity, thereby increasing the viscoelastic contribution to the Cauchy stress, magnified the differences between the hyperelastic and viscoelastic cases (essentially magnifying the effects seen in Fig. 12). Additional simulations (not reported here) also showed that higher values of $\delta$ not only reduced the magnitude of $\varvec{\varPi }$, $\varvec{u}$, *p*, but also smoothed their profiles, reducing the distances between peaks and troughs. Even if no substantial differences were encountered in terms of conduction velocity, the calcium transients displayed generally higher values in the viscoelastic case.

### Viscoelastic versus hyperelastic effects under passive inflation and active contraction

Much more evident differences can be observed in terms of the true stress $\sigma _f = \mathbf {F}{\varvec{f}_0}\cdot (\varvec{\sigma }\mathbf {F}{\varvec{f}_0})$ when plotted against the local stretch in the fibre direction, $\lambda _f=\sqrt{I_{4,f}}$. Such a comparison has been conducted in Gültekin et al. (2016) for idealised geometries, and it was specifically designed to study hysteresis effects due to viscous contributions to orthotropic passive stress. For the inflation tests, we will restrict to $\beta = 1$ ms and $\delta = 22.6$ N/cm$^2\cdot$ms. These values, considered in Karlsen (2017) (and using units of [s] and [Pa s], respectively), ensure that the viscoelastic component is large enough to have a visible effect, but does not completely overwhelm the dynamics of the tissue. Here, we consider the left ventricular domain used in Sect. 4.5 and proceed to analyse a stress–stretch response on two points near the basal surface on the endocardium and epicardium, portrayed in Fig. 13a. The mechanical parameters were taken differently from those in Table 2; here, we focus on the patient-specific constants estimated from healthy myocardial tissue at 8 mmHg end-diastolic pressure using chamber pressure-volume and strain data taken in vivo (Gao et al. 2015). The modified values for this particular test are $a=0.02096$ N/cm$^2$, $b=3.243$, $a_f=0.30634$ N/cm$^2$, $b_f=3.4595$, $a_s=0.07334$ N/cm$^2$, $b_s=1.5473$, $a_{fs}=0.03646$ N/cm$^2$, $b_{fs}=3.39$, and we set $\zeta _{\text {stab}} = 10$. In the simulation, we impose a sinusoidal endocardial pressure of maximal amplitude 0.1 N/cm$^2$ (approximately 8 mmHg) and run a set of transient simulations over the interval from 0 to 300 ms. This configuration constitutes an inflation and deflation process where the majority of the fibres are acting in traction, whereas sheetlets work under a compression regime. Plots (b, c) in Fig. 13 illustrate the stress–stretch response (in terms of the true stress). The behaviour on the epicardial point shows an exponential stiffening and is quite similar to what was observed in Gültekin et al. (2016), as for both stress measures in the viscoelastic case there is evidence of hysteresis effects (that are, by definition, not present in the hyperelastic case). Slight deviations from the reference results in Gültekin et al. (2016) may be related to the fact that we are using a full electromechanical model, a different viscoelastic contribution, and different material parameters. On the endocardial point, we observe even more marked differences between the two cases, probably since we do not expect symmetry in the motion patterns for a non-ellipsoidal geometry. Other qualitative differences in the motion patterns include a more marked wall thickening, and an overall lower pressure (also more evenly spread throughout the endocardium, showing a smoother profile than the one produced in the hyperelastic case). Pressure on the epicardium was higher in the viscoelastic case.

**Fig. 14:**
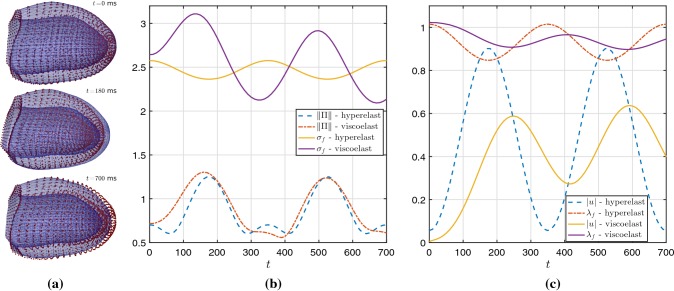
Comparison between hyperelastic and viscoelastic effects under contraction, using the active strain approach and $\beta = 5$ ms and $\delta = 80.25$ N/cm^2^ ms. Panel **a** has side views of the deformed domain for viscoelastic (hollow blue) and hyperelastic (dark red dots) at three different times, and panels **b**, **c** show transient of mechanical outputs extracted from a point on the lower epicardial surface

As a final test, we analyse the differences between the viscoelastic and hyperelastic case under active contraction. We employ the ventricular geometry again, imposing Robin boundary conditions on the epicardial surface and zero normal displacements on the basal cut, and use the active strain approach. We use $\zeta _{\text {stab}} = 10$, and more pronounced viscoelastic effects encoded in $\beta = 5$ ms and $\delta = 80.25$ N/cm^2^ ms. The active contraction of the ventricle is initiated by an ectopic beat and impose a sinusoidal endocardial pressure but with larger amplitude 0.25 N/cm$^2$ and simulate the process for approximately two cycles (from 0 to 700 ms). Figure 14 reveals a higher asynchrony in the tissue deformation and stresses between the hyperelastic and viscoelastic case. This is observed visibly from the motion of the ventricle in panel (a), but also from the transients extracted on an epicardial point near the apex and measuring true stress in the fibre direction, the local fibre stretch, the displacement magnitude. This effect was milder in the Frobenius norm of the Kirchhoff stress tensor.

## Concluding remarks

We have introduced a model for the active contraction of cardiac tissue. We focused on incorporating the mechanoelectric feedback through stress-assisted diffusion, accounting for a porous-media-type nonlinear diffusivity, and including inertial terms in the equations of motion. The three-field equations of motion of a viscoelastic orthotropic material are coupled with a four-variable minimal model for human ventricular action potential using both active strain and active stress approaches. We have also proposed a new stabilised mixed-primal numerical scheme written, in particular, in terms of the Kirchhoff stress. The non-trivial effects of both viscoelasticity and stress-assisted diffusion in our model suggest that they may play an important role in governing cardiac function and its response to external stimuli.

An important remark is that the active strain approach seems to be much more robust than the active stress formulation, at least in the present context. In order to obtain comparable results with the active stress approach, we had to spend quite a lot of effort finding the correct scaling in (2.3). Once this is achieved, we observed that there is no substantial difference in the output quantities. This is why our numerical tests have focused a little bit more on the active stress, since it is somewhat more challenging than the other case.

Further additions will be mostly focused on multiscale microstructural coupling, which will provide a more physiological justification of the model in terms of complex phenomena involved in mechanoelectrical interactions. One example would be to include poroelastic effects representing perfusion of the myocardial tissue. Developing a thermodynamically consistent description of stress-assisted diffusion is also a pending task, in which electromechanical coupling with the surrounding torso and organs would represent another level of interaction. Such formulation under electromechanical coupling (and including nonlinear and stress-assisted diffusion) will require state-of-the-art tools of multiscale homogenisation (Cyron et al. 2016) as well as dedicated multiscale numerical methods (Gandhi and Roth 2016).

More confident now in obtaining accurate and reliable numerical solutions, our forthcoming contributions will target an exhaustive computational analysis of restitution curves and realistic activation patterns, e.g. accounting for Purkinje fibres and cellular heterogeneity, with the purpose of characterising spatiotemporal alternans patterns (Gizzi et al. 2013) in the presence of multiple mechanoelectric feedback effects. Practical applications of the present study rely on the antitachycardia pacing protocols, as well as on the (still today not completely understood) effects of mechanical loads, including cardiac massage, tissue damage, and remodelling at different scales during atrial flutter (Masé et al. 2008). Estimates of energy dissipation and heat production would be further investigated, widening the validity of these models to non-equilibrium thermodynamical systems. The simulation of mechanically induced ectopic activity, as well as prediction of the risk of sudden cardiac death, is also part of our long-term goals. For the sake of model validation, we are also interested in marrying our results to experimental observations obtained through elastography, using, for instance, the novel approach advanced in Capilnasiu et al. (2019).

## Details on the minimal ionic model

The ionic currents consist of three general terms, phenomenologically constructed (without particularisation to the ionic species that generate them)

$$\begin{aligned} g(v,\vec {r})&= g^{\text {fi}}(v,\vec {r}) + g^{\text {si}}(v,\vec {r}) + g^{\text {so}}(v,\vec {r}), \end{aligned}$$

where the adimensional *fast inward*, *slow inward*, and *slow outward* currents are, respectively, given by

$$\begin{aligned} \chi \, g^{\text {fi}}(v,\vec {r})&= -r_1 \mathcal {H}(v-\theta _1)(v-\theta _1)(v_v-v)/\tau _{fi}, \\ \chi \, g^{\text {si}}(v,\vec {r})&= -\mathcal {H}(v-\theta _2) r_2 r_3/\tau _{si}, \\ \chi \, g^{\text {so}}(v,\vec {r})&= \frac{(v-v_0)(1-\mathcal {H}(v-\theta _2))}{\tau _o}+\mathcal {H}(v-\theta _2)/\tau _{so}, \end{aligned}$$

and the kinetics of the gating variables $\vec {r}$ are given by

$$\begin{aligned}&\vec {m}(v,\vec {r})=\\&\begin{pmatrix} (1-\mathcal {H}(v-\theta _1))(r_{1,\inf } - r_1)/\tau _1^- - \mathcal {H}(v-\theta _1)r_1/\tau _1^+\\ (1-\mathcal {H}(v-\theta _2))(r_{2,\inf } - r_2)/\tau _2^--\mathcal {H}(v-\theta _2)r_2/\tau _2^+\\ ((1+\tanh (k_3(v-v_3)))/2-r_3)/\tau _3 \end{pmatrix}. \end{aligned}$$

Here, $\mathcal {H}$ is the Heaviside step function, and the time constants and steady-state values are defined as:

$$\begin{aligned} \tau _1^-&=(1-\mathcal {H}(v-\theta _1^-))\tau _{1,1}^-+\mathcal {H}(v-\theta _1^-)\tau _{1,2}^-,\\ \tau _2^-&=\tau _{2,1}^-+(\tau _{2,2}^--\tau _{2,1}^-)(1+\tanh (k_2^-(v-v_2^-)))/2,\\ \tau _{so}&=\tau _{so,1}+(\tau _{so,2}-\tau _{so,1})(1+\tanh (k_{so}(v-v_{so})))/2,\\ \tau _3&=((1-\mathcal {H}(v-\theta _2))\tau _{3,1}+\mathcal {H}(v-\theta _2)\tau _{3,2}, \\ \tau _o&= ((1-\mathcal {H}(v-\theta _0))\tau _{o,1}+\mathcal {H}(v-\theta _0)\tau _{o,2},\\ r_{1,\inf }&= {\left\{ \begin{array}{ll} 1, &{} v<\theta _1^-\\ 0,&{} u\ge \theta _1^-\end{array}\right. },\\ r_{2,\inf }&= ((1-\mathcal {H}(v-\theta _0))(1-v/\tau _{2,\infty })+\mathcal {H}(v-\theta _0) r_{2,\infty }^*. \end{aligned}$$

## Mixed-primal fully discrete finite element scheme

We restrict the presentation to the active stress formulation using a smoothed model for active tension (stressing that the case of active strain and other boundary conditions follows a similar treatment). Let us denote by $\mathcal {T}_h$ a regular partition of $\varOmega$ into simplicial elements *K* (pairwise disjoint triangles in 2D or tetrahedra in 3D) of maximum diameter $h_K$, and define the mesh size as $h:=\max \{h_K: K\in \mathcal {T}_h\}$. Let us also denote by $\mathcal {E}_h$ the set of interior facets of the mesh, and by $[\![\cdot ]\!]_e$ the jump of a quantity across a given facet $e\in \mathcal {E}_h$. The specific finite element method we use here is based on solving the discrete weak form of the hyperelasticity equations using piecewise constant approximations of the symmetric Kirchhoff stress tensor, piecewise linear approximation of displacements, and piecewise constant approximation of (solid) pressure. The transmembrane potential in the electrophysiology equations is discretised with Lagrange finite elements (piecewise linear and continuous functions), and the remaining ionic quantities are approximated by piecewise constant functions. More precisely, we use the finite-dimensional spaces $\mathbb {H}_h\subset \mathbb {L}^2_{\text {sym}}(\varOmega )$, $\mathbf {V}_h\subset \mathbf {H}^1(\varOmega )$, $W_h\subset \mathrm {H}^1(\varOmega )$, $Q_h\subset \mathrm {L}^2(\varOmega )$, $Z_h\subset \mathrm {L}^2(\varOmega )^3$ defined (for the case of a generic-order approximation $l\ge 0$) as follows:

$$\begin{aligned} \mathbb {H}_h&:=\{ \varvec{\tau }_h\in \mathbb {L}^2_{\text {sym}}(\varOmega ) : \varvec{\tau }_h|_K\in \mathbb {P}_l(K)^{d\times d},\forall K\in \mathcal {T}_h\},\\ \mathbf {V}_h&:=\{\varvec{v}_h\in \mathbf {H}^1(\varOmega ):\varvec{v}_h|_K\in \mathbb {P}_{l+1}(K)^d,\forall K\in \mathcal {T}_h,\\&\qquad \qquad \varvec{v}_h\cdot \varvec{n}=0 \text { on } \partial \varOmega _D\},\\ Q_h&:= \{ q_h\in \mathrm {L}^2(\varOmega ) : q_h|_K\in \mathbb {P}_{l}(K),\forall K\in \mathcal {T}_h\},\\ W_h&:= \{ w_h\in \mathrm {H}^1(\varOmega ) : w_h|_K\in \mathbb {P}_{l+1}(K),\forall K\in \mathcal {T}_h\},\\ Z_h&:= \{ \varphi _h\in \mathrm {L}^2(\varOmega ) : \varphi _h|_K\in \mathbb {P}_{l}(K),\forall K\in \mathcal {T}_h\}, \end{aligned}$$

where $\mathbb {P}_l(K)$ denotes the space of polynomial functions of degree $s\le l$ defined locally on the element *K*. Assuming zero body loads, and applying a backward differentiation formula (BDF) for the time integration, we end up with the following fully discrete nonlinear electromechanical problem, starting from the discrete initial data $v^0_h,n^{0}_h,T_{a,h}^0$. For each $n=0,1,\ldots ,n_{\max }$: find

$(\varvec{\varPi }_h^{n+1},\varvec{u}_h^{n+1},p_h^{n+1})$ and $(v_h^{n+1},\vec {r}_h^{n+1},T_{a,h}^{n+1})$ such that

B.1 $$\begin{aligned}&\int _\varOmega [\varvec{\varPi }_h^{n+1} - \mathcal {G}(\varvec{u}_h^{n+1}) + p_h^{n+1}J(\varvec{u}_h^{n+1})\mathbf {I}] : \varvec{\tau }_h = 0 \qquad \forall \varvec{\tau }_h \in \mathbb {H}_h, \\&\int _\varOmega \frac{\varvec{u}_h^{n+1}-2\varvec{u}_h^n+\varvec{u}_h^{n-1}}{\varDelta t^2}\cdot \varvec{v}_h +\int _\varOmega \varvec{\varPi }_h^{n+1}\! :\! \nabla \varvec{v}_h \mathbf {F}^{\mathtt{-t}}(\varvec{u}_h^{n+1}) \\&\quad - \int _{\partial \varOmega _N} \!\!p_N \mathbf {F}^{\mathtt{-t}}(\varvec{u}_h^{n+1}) \varvec{n}\cdot \varvec{v}_h = 0 \qquad \forall \varvec{v}_h \in \mathbf {V}_h, \\&\int _\varOmega [J(\varvec{u}_h^{n+1}) - 1 ] q_h + \sum _{e\in \mathcal {E}_h} \int _e \zeta _{\text {stab}}h_e [\![p_h^{n+1}]\!]_e [\![q_h]\!]_e = 0 \qquad \forall q_h\in Q_h, \\&\int _\varOmega \frac{v_h^{n+1}-v_h^{n}}{\varDelta t} w_h + \int _\varOmega \mathbf {D}(v_h^{n+1},\varvec{\varPi }_h^{n+1})\, \nabla v_h^{n+1} \cdot \nabla w_h \\&\quad - \int _\varOmega \biggl [g(v_h^{n},\vec {r}_h^{n}) + I_{\mathrm{ext}}\biggr ] w_h = 0 \qquad \forall w_h \in W_h, \\&\int _\varOmega \frac{\vec {r}_h^{n+1}-\vec {r}_h^{n}}{\varDelta t} \cdot \vec {s}_h - \int _\varOmega \vec {m}(v_h^{n},\vec {r}_h^{n})\cdot \vec {s}_h = 0 \qquad \forall \vec {s}_h \in Z_h^3, \\&\int _\varOmega \frac{T_{a,h}^{n+1}-T_{a,h}^{n}}{\varDelta t} \varphi _h + \alpha _1D_0\int _\varOmega \nabla T_{a,h}^{n+1} \cdot \nabla \varphi _h \\&\quad - \int _\varOmega \ell (T_{a,h}^{n+1},\vec {r}_h^{n}) \varphi _h = 0 \qquad \forall \varphi _h\in W_h, \end{aligned}$$

where $\zeta _{\text {stab}}$ is a positive pressure stabilisation parameter required to enforce solvability of the discrete problem. This is the tetrahedral counterpart of the finite element method for quadrilateral meshes studied in Chavan et al. (2007) and recently used in the context of cardiac electromechanics in Ruiz-Baier et al. (2019). Notice that the boundary condition (2.10a) is incorporated as an essential condition on the displacement space, whereas the traction boundary condition (2.10b) on the remainder of the boundary $\partial \varOmega _N$ appears naturally as the last term in the second equation of (B.1).

## Linearisation of the mechanical problem

The coupling between activated mechanics and the electrophysiology solvers will be performed using a segregated fixed-point scheme. At each time step, the nonlinear algebraic sub-system for the mechanics defined by the first three equations in (B.1) is linearised, adopting the following form (where the time indices have been dropped for the sake of notation).

Starting from the initial guess $(\varvec{\varPi }_h^{k=0},\varvec{u}_h^{k=0},p_h^{k=0}) = (\varvec{\varPi }_h^n,\varvec{u}_h^n,p_h^n)$, for $k=0,1,\ldots$ find stress, displacement, and pressure increments $(\delta \varvec{\varPi }_h^{k+1},\delta \varvec{u}_h^{k+1},\delta p_h^{k+1})$ such that

C.1 $$\begin{aligned}&\int _\varOmega \bigl [\delta \varvec{\varPi }_h^{k+1} + \frac{\partial \mathcal {G}_h^k}{\partial \mathbf {F}_h^k}\nabla \delta \varvec{u}_h^{k+1} \\&\quad +(\delta p_h^{n+1}J_h^{k} + p_h^kJ_h^k\mathbf {F}_h^{k,\mathtt{-t}}:\nabla \delta \varvec{u}_h^{k+1})\mathbf {I}\bigr ] : \varvec{\tau }_h \\&\quad = \int _\varOmega \mathcal {R}^k_{\varvec{\varPi }}:\varvec{\tau }_h \qquad \forall \varvec{\tau }_h \in \mathbb {H}_h, \\&\int _\varOmega \frac{\delta \varvec{u}_h^{k+1}}{\varDelta t^2}\cdot \varvec{v}_h \\&\quad +\int _\varOmega \bigl [\delta \varvec{\varPi }_h^{k+1}\mathbf {F}_h^{k,\mathtt{-t}}-\varvec{\varPi }_h^{k}\mathbf {F}_h^{k,\mathtt{-t}}(\nabla \delta \varvec{u}_h^{k+1})^\mathtt{t} \mathbf {F}_h^{k,\mathtt{-t}}\bigr ] : \nabla \varvec{v}_h \\&\quad + \int _{\partial \varOmega _N} \!\!p_N \mathbf {F}_h^{k,\mathtt{-t}}(\nabla \delta \varvec{u}_h^{k+1})^\mathtt{t} \mathbf {F}_h^{k,\mathtt{-t}}\varvec{n}\cdot \varvec{v}_h \\&\quad = \int _\varOmega \varvec{\mathcal {R}}^k_{\varvec{u}}\cdot \varvec{v}_h \qquad \forall \varvec{v}_h \in \mathbf {V}_h, \\&\int _\varOmega \bigl (J_h^k\mathbf {F}_h^{k,\mathtt{-t}}:\nabla \delta \varvec{u}_h^{k+1}\bigr ) q_h \\&\quad + \sum _{e\in \mathcal {E}_h} \int _e \zeta _{\text {stab}}h_e [\![ \delta p_h^{n+1}]\!]_e[\![q_h]\!]_e = \int _\varOmega \mathcal {R}^k_{p}q_h \qquad \forall q_h\in Q_h, \end{aligned}$$

and then update $\varvec{\varPi }_h^{k+1} = \varvec{\varPi }_h^k + \delta \varvec{\varPi }_h^{k+1}$, $\varvec{u}_h^{k+1} = \varvec{u}_h^k + \delta \varvec{u}_h^{k+1}$, $p_h^{k+1} = p_h^k + \delta p_h^{k+1}$. Here, $\mathcal {R}^k_{\varvec{\varPi }}, \varvec{\mathcal {R}}^k_{\varvec{u}},\mathcal {R}^k_{p}$ are tensor, vector, and scalar residuals associated with the Newton–Raphson linearisation at the previous step *k*, and $\mathbf {F}_h^k=\mathbf {I}+ \nabla \varvec{u}_h^k$, $J_h^{k} = \det \mathbf {F}_h^k$. Next we introduce the following linear maps (related to the Gâteaux derivatives of the solution operator)

$$\begin{aligned} \mathcal {L}_1^k: \mathbb {H}_h \rightarrow \mathbb {H}_h,&\varvec{\tau }\mapsto \mathcal {L}_1^k (\varvec{\tau }) = \frac{\partial \mathcal {G}_h^k}{\partial \mathbf {F}_h^k} \varvec{\tau }\!+\! (p_h^kJ_h^k\mathbf {F}_h^{k,\mathtt{-t}}\!\!:\varvec{\tau }) \mathbf {I},\\ \mathcal {L}_2^k: \mathbb {H}_h \rightarrow \mathbb {H}_h,&\varvec{\tau }\mapsto \mathcal {L}_2^k (\varvec{\tau }) =- \varvec{\varPi }_h^{k}\mathbf {F}_h^{k,\mathtt{-t}} \varvec{\tau }^\mathtt{t} \mathbf {F}_h^{k,\mathtt{-t}}, \end{aligned}$$

as well as the bilinear forms and linear functionals

$$\begin{aligned} A_1(\varvec{\varPi },\varvec{\tau })&= \int _\varOmega \varvec{\varPi }: \varvec{\tau }, \\ A_2 (\varvec{u},\varvec{v})&= \frac{1}{\varDelta t^2} \!\! \int _\varOmega \varvec{u}\cdot \varvec{v}+ \int _\varOmega \mathcal {L}_2^k(\nabla \varvec{u}):\nabla \varvec{v}, \\ A_3(p,q)&= \sum _{e\in \mathcal {E}_h}\!\! \int _e \zeta _{\text {stab}}h_e [\![ p]\!]_e\![\![q]\!]_e, \\ B_1(\varvec{u},\varvec{\tau })&= \int _\varOmega \mathcal {L}_1^k(\nabla \varvec{u}) : \varvec{\tau },\\ \widetilde{B}_1(\varvec{\varPi },\varvec{v})&= \int _\varOmega \varvec{\varPi }\,\mathbf {F}_h^{k,\mathtt{-t}} :\nabla \varvec{v}, \\ B_2(p,\varvec{\tau })&= \int _\varOmega J_h^k\, p\mathop {\mathbf {tr}}\nolimits (\varvec{\tau }), \\ \widetilde{B}_3(\varvec{u},q)&= \int _\varOmega (J_h^k\mathbf {F}_h^{k,\mathtt{-t}}\!:\!\nabla \varvec{u})\,q, \ F_1(\varvec{\tau }) = \int _\varOmega \mathcal {R}^k_{\varvec{\varPi }}:\varvec{\tau },\\ F_2(\varvec{v})&= \int _\varOmega \varvec{\mathcal {R}}^k_{\varvec{u}}\cdot \varvec{v},\quad F_3(q) = \int _\varOmega \mathcal {R}^k_{p}q. \end{aligned}$$

Then, dropping all iteration indices and making abuse of notation, the tangent problem (C.1) (now also restricted to pure displacement boundary conditions) can be recast as the mixed variational form

C.2 $$\begin{aligned} A_1(\varvec{\varPi }_h,\varvec{\tau }_h) + B_1(\varvec{u}_h,\varvec{\tau }_h) +\ B_2(p_h,\varvec{\tau }_h)&= F_1(\varvec{\tau }_h) \forall \varvec{\tau }_h \in \mathbb {H}_h, \\ \widetilde{B}_1(\varvec{\varPi }_h,\varvec{v}_h) + A_2 (\varvec{u}_h,\varvec{v}_h)&= F_2(\varvec{v}_h) \forall \varvec{v}_h \in \mathbf {V}_h, \\ \widetilde{B}_3(\varvec{u}_h,q_h) + A_3(p_h,q_h)&= F_3(q_h) \,\,\forall q_h \in Q_h. \end{aligned}$$

The stability and convergence analysis of (C.2) will be studied in a forthcoming contribution.
